# Inorganic Arsenic-induced cellular transformation is coupled with genome wide changes in chromatin structure, transcriptome and splicing patterns

**DOI:** 10.1186/s12864-015-1295-9

**Published:** 2015-03-19

**Authors:** Caitlyn Riedmann, Ye Ma, Manana Melikishvili, Steven Grason Godfrey, Zhou Zhang, Kuey Chu Chen, Eric C Rouchka, Yvonne N Fondufe-Mittendorf

**Affiliations:** Department of Molecular and Cellular Biochemistry, University of Kentucky, Lexington, KY 40536 USA; Graduate Center for Toxicology, University of Kentucky, Lexington, KY 40536 USA; Department of Pharmacology and Nutritional Sciences, University of Kentucky, Lexington, KY 40536 USA; Department of Computer Engineering and Computer Science, University of Louisville, Louisville, KY 40292 USA

**Keywords:** Gene expression, Genome-wide, Chromatin, Arsenic, Splicing

## Abstract

**Background:**

Arsenic (As) exposure is a significant worldwide environmental health concern. Low dose, chronic arsenic exposure has been associated with a higher than normal risk of skin, lung, and bladder cancer, as well as cardiovascular disease and diabetes. While arsenic-induced biological changes play a role in disease pathology, little is known about the dynamic cellular changes resulting from arsenic exposure and withdrawal.

**Results:**

In these studies, we sought to understand the molecular mechanisms behind the biological changes induced by arsenic exposure. A comprehensive global approach was employed to determine genome-wide changes to chromatin structure, transcriptome patterns and splicing patterns in response to chronic low dose arsenic and its subsequent withdrawal. Our results show that cells exposed to chronic low doses of sodium arsenite have distinct temporal and coordinated chromatin, gene expression, and miRNA changes consistent with differentiation and activation of multiple biochemical pathways. Most of these temporal patterns in gene expression are reversed when arsenic is withdrawn. However, some gene expression patterns remained altered, plausibly as a result of an adaptive response by cells. Additionally, the correlation of changes to gene expression and chromatin structure solidify the role of chromatin structure in gene regulatory changes due to arsenite exposure. Lastly, we show that arsenite exposure influences gene regulation both at the initiation of transcription as well as at the level of splicing.

**Conclusions:**

Our results show that adaptation of cells to iAs-mediated EMT is coupled to changes in chromatin structure effecting differential transcriptional and splicing patterns of genes. These studies provide new insights into the mechanism of iAs-mediated pathology, which includes epigenetic chromatin changes coupled with changes to the transcriptome and splicing patterns of key genes.

**Electronic supplementary material:**

The online version of this article (doi:10.1186/s12864-015-1295-9) contains supplementary material, which is available to authorized users.

## Background

Arsenic, a ubiquitous metalloid, is one of the most common environmental pollutants, with human exposure occurring mainly through contaminated drinking water. In some regions of the world, especially coal mining regions, inorganic arsenic (iAs) levels in drinking water can exceed those recommended by the World Health Organization [1,2]. Long-term exposure to iAs is associated with the etiology of several diseases including coronary heart disease, hypertension, arteriosclerosis and multiple cancers [3-5]. Although arsenic is a recognized human carcinogen, the mechanism(s) by which it causes cancer remains elusive.

Arsenic is found in several different chemical forms and oxidation states and its metabolism has an important role in its toxicity. In mammals, the metabolism of arsenic is catalyzed by Arsenic (+3 oxidation state) methyltransferase 1 (AS3MT 1) which catalyzes conversion of iAs to methylated arsenicals. This process involves a sequential reduction of iAs^5+^ to iAs^3+^ followed by oxidative methylation to monomethylarsonic acid (MMA^5+^) and dimethylarsinic acid (DMA^5+^). Some of the intermediates of this process, the trivalent intermediate arsenicals, MMA^3+^ and DMA^3+^, have been implicated in arsenic toxicity, acting as potent cytotoxins and enzyme inhibitors. Arsenic thus causes oxidative stress, apoptosis and mutagenesis, all mechanisms important for its carcinogenic potential [reviewed in [6]. However, since arsenic does not cause point mutations [7,8], other mechanisms have been implicated in its toxicity. Accumulating evidence suggests that aberrant gene expression due to non-genotoxic modifications may play crucial roles in arsenic-mediated carcinogenesis [9-11]. Furthermore the lack of a prominent signal transduction mechanism has led to the belief that arsenic is an epigenetic carcinogen. Nonetheless, signal transduction pathways can integrate arsenic-induced signals into specific transcriptional states, characterized by chromatin structures that activate or repress transcription at specific gene loci. Although a large variety of signal transduction pathways have already been described, much less is known about the crosstalk between signal transduction and the consequent changes in chromatin structure that lead to changes in gene expression.

In order to understand the mechanism of iAs toxicity several microarray-based gene expression studies have been conducted [3]. These studies have shown widespread disruption of transcriptional activity following iAs exposure with extensive changes in global gene expression, suggesting that diverse regulatory mechanisms of gene expression might be affected. However, most of these studies have analyzed gene expression changes caused by acute phase responses and instant adaptation of cells to iAs insult. Lacking are comprehensive studies on low-dose, long-term iAs exposure. Such studies will have a fundamental impact on our understanding of arsenic-disease developmental changes. Equally important and lacking is an understanding of whether iAs-induced gene expression changes are reversible upon removal of the toxic insult, and if so to what extent. Lastly, it remains to be determined whether iAs influences mRNA splicing patterns.

Arsenic is known to transform cells through the epithelial-to-mesenchymal transition (EMT) [12,13]. Although arsenic-regulated expression of individual genes has been intensively studied, the biological consequences of global chromatin, transcriptome and splicing changes caused by this metal during EMT remain unexplored. To increase our understanding of the underlying molecular mechanisms of iAs-induced EMT, we investigated the consequences of protracted iAs exposure and its subsequent withdrawal on chromatin structural changes, gene expression and alternative splicing. Our findings demonstrate that the adaptational changes due to iAs exposure involve changes to chromatin structure, gene expression as well as the production of specific gene isoforms. Additionally, withdrawal of iAs results in the restoration of some, but not all, chromatin structures and gene expression patterns. The permanent alteration of some gene expression patterns possibly could be linked with disease etiology associated with arsenic exposure. Additionally most of these patterns were consistent in both HeLa and BEAS-2B cells, suggesting some pathways modulated by iAs might be universal.

## Results

### Exposure to low doses of sodium arsenite suppresses cell growth and modulates cell morphology

**Figure 1:**
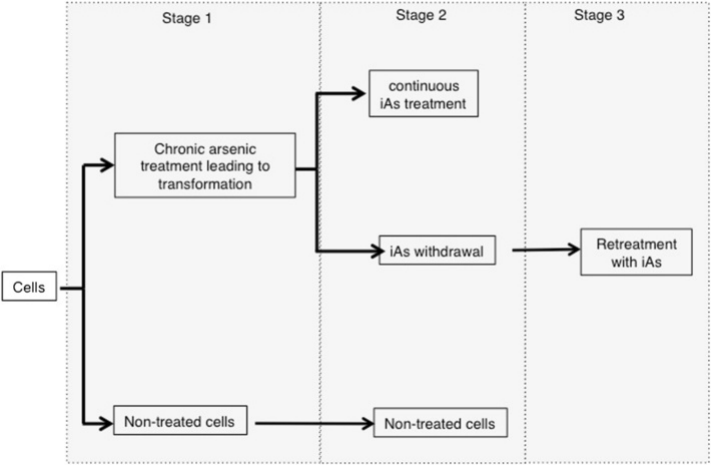
**Experimental design.** Cells were assigned to 4 treatment groups: control (NT), chronic exposure and transformed (iAs-T), withdrawal (iAs-Rev-cells were treated for 26 days with iAs, then iAs was removed and cells grown for another 10 days) or retreatment (same as iAs-Rev but after these 10 days, cells were retreated with iAs for another 10 days). This latter experiment tests for the genes altered immediately by iAs-exposure. These groups were chosen based on observations to changes in cell morphology. Furthermore, these time points are intended to capture transcription changes due to long term arsenic exposure and withdrawal.

To understand the effect of long-term iAs exposure on cells resulting in EMT, we carried out studies in normal lung epithelial BEAS-2B cells, bearing in mind that the lung is a major cellular target of iAs carcinogenesis [4,14-18]. In addition to normal BEAS-2B cells, we also used the cervical cancer cell line, HeLa, a commonly used model cell line to study cancer cell signaling and EMT. We used these cells lines to assess whether the observed iAs-mediated effects were applicable to both normal and malignant cells. We initially investigated the effect of sodium arsenite (iAs^3+^) on the proliferation of these cells using environmentally relevant doses of ≤1 μM [19,20]. Higher concentrations of iAs were briefly examined and as expected led to consistent cell death. Our experimental design is described in Figure 1.

**Figure 2:**
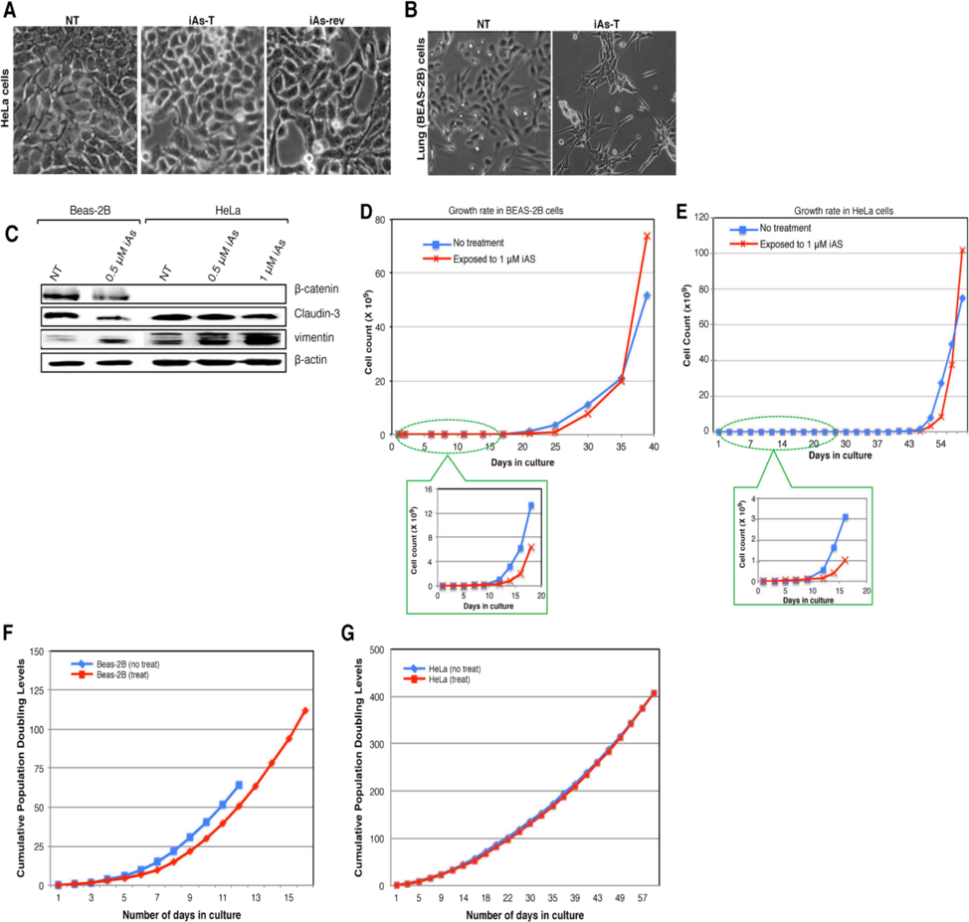
**Chronic low dose exposure of sodium arsenite changed cell morphology. A)** In HeLa cells **B)** In BEAS-2B cells after 30 days in culture, starting to show some elongated characteristics and at 36 days showed elongated, fibroblast-like shape. Phase-contrast microscopy observed the morphological changes of HeLa and BEAS-2B cells. All images were obtained at a magnification of × 100. **C)** Confirmation of EMT transition using western blot analyses of known EMT markers, claudin-3, Vimentin and β-catenin. **D - G**: Chronic exposure to low dose iAs-exposure alters cell growth. Proliferation of non-treated and cells treated with inorganic arsenite continuously for several weeks. Growth rate curves were generated by growing cells and counting them every 2-3 days at 80% confluency. Growth curve of NT and iAs-treated cells **D)** BEAS 2B and **E)** HeLa cells. Data are mean S.E.M. of 3 independent experiments; P < 0.01. Sodium arsenite extends lifespan of **F)** BEAS-2B and **G)** HeLa cells. Cumulative population doubling curves of BEAS-2B cells (left) and HeLa (right) in normal growth medium (blue lines) or cells exposed to 0.5 μM sodium arsenite (red lines). Viable cells were counted weekly by trypan blue staining using a hemacytometer. Population doublings were calculated by the formula log [(number of cells harvested)/(number of cells seeded)]/log2. Each graph depicts the averaged results from three longevity assessments.

Cells were cultured with and without 0.5 μM and 1 μM iAs for 16 weeks (Figure 1), during which time we monitored their morphology and growth rates (Figure 2). In “phase 1” (36 days for BEAS-2B and 45 days for HeLa), iAs-treated cells showed slow growth compared to time-matched non-treated (NT) cells (Figures 2A – D). These data are consistent with previous studies that showed inhibition of cell growth due to iAs exposure [12]. In “phase II” (after 36 days for BEAS-2B and 45 days for HeLa), changes in growth and cell morphology became apparent as iAs-treated cells grew faster than NT cells and even surpassed their non-exposed counterparts (Figures 2A - D). HeLa cells became rounder, grew on top of each other and lost their filapodia (Figure 2A), while BEAS-2B cells became more fibroblast-like (Figure 2B). These results are comparable with reported malignant transformations by inorganic arsenic in other cell lines [21]. We hypothesize that these changes in cell morphology and increased growth rate in both cell-types indicate a point of transformation of iAs-treated cells possibly undergoing an EMT.

To confirm that indeed we are observing an EMT, we examined whether the expression of EMT markers were concomitantly altered. Cell lysates were probed using antibodies against vimentin and claudin-3, known EMT markers, in Western blot analyses. We found that claudin-3 protein levels were downregulated by ~65% in iAs-exposed cells. On the other hand, vimentin protein levels were highly induced by iAs exposure (40% increase compared to NT cells in both cell types). Interestingly removal of arsenite resulted in a reversal of these changes in protein levels, additionally, this effect was dose dependent as we observed more expression of vimentin in 1 μM compared to 0.5 μM arsenite treatment (Figure 2C and Additional file 1: Figure S1). Henceforth, we will refer to these cells as iAs-transformed (iAs-T) cells.

### Arsenic-exposure results in an increased lifespan of cells

To determine the effect of iAs on population-doubling capacity, BEAS-2B and HeLa cells were continuously cultured in growth media containing 1 μM iAs. Exposure of the HeLa cells to iAs did not have an effect on the cell proliferation rate as the cells doubled as fast as NT cells (Figure 2E). We attribute this to the fact that these cells are carcinogenic. However treated BEAS-2B had a slower population-doubling rate and a longer *in vitro* lifespan compared to NT cells (Figure 2F). These iAs-treated BEAS-2B cells grew continuously without a detectable senescent phenotype [13], possibly mirroring iAs transformation of these cells. Taken together, our results support the idea that signal transduction mechanisms elicited by low doses of iAs exposure and subsequent induction of defense mechanisms contribute to longevity.

### Low doses of iAs does not induce DNA fragmentation

In order to determine if a chronic low dose of iAs exposure results in apoptosis, we tested for genomic DNA laddering, a well characterized marker for apoptosis [22]. DNA from the NT, 0.5 μM and 1 μM iAs-treated cells (both BEAS-2B and HeLa cells) was purified and analyzed using agarose gel electrophoresis. We found that chronic low doses of iAs did not induce DNA fragmentation in these cells (Additional file 2: Figure S2). While our bulk studies do not exclude the possibility of some level of apoptosis occurring, they suggest that other mechanisms are likely responsible for the gene expression changes observed in iAs-induced cellular transformation. One likely mechanism for the changes in gene expression observed in iAs-transformed cells is modulation to their epigenome.

### Low doses of arsenite induce structural changes to chromatin

Differentiating cells undergo programmed alterations in their patterns of gene expression, which are often regulated by structural changes in chromatin. We therefore asked if iAs induces changes to chromatin structure during the process of iAs-mediated cellular transformation. To this end, we used several methods to test for chromatin structural changes - bulk nucleosome repeat length (NRL), micrococcal (MNase) resistance and the presence of the repressive histone H1.

**Figure 3:**
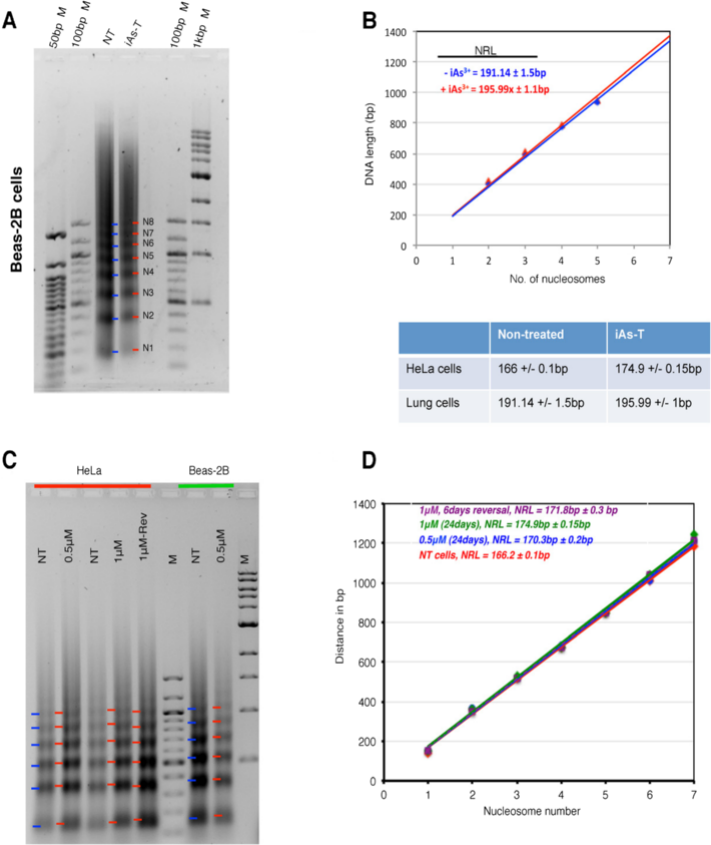
**Arsenite treatment causes a reduction in nucleosome spacing. A)** Nuclei from control and arsenic-transformed BEAS-2B cells were treated with MNase and the profile of bulk chromatin was analyzed by gel electrophoresis to calculate the nucleosome repeat length (NRL). **B)** Plot of nucleosome number versus DNA length for MNase-digested bulk chromatin of BEAS-2B cells ± arsenic. This plot was used to calculate the corresponding NRL. Data from 3 independent experiments has been pooled and the average ± SD is shown. Blue bars and red bars correspond to nucleosome ladders from NT and iAs-T cells respectively. **C)** Similar studies were done in HeLa cells and **D)** NRL was calculated in NT, iAs-T and iAs-Rev cells.

For bulk NRL changes, we isolated nuclei from NT and iAs-T cells to ensure maximal effect of arsenite treatment. Chromatin was digested *in situ* with different concentrations of MNase, and the resulting partially digested DNA fragments resolved by agarose gel electrophoresis (Figure 3A). NRL an indication of chromatin compactness was measured according to Nalabothulla *et al*, [23]. A single basepair change in NRL, could allow for a transcription factor-binding site previously occluded by the nucleosome to become available, resulting in changes in transcriptional outcomes [23,24]. Measuring the NRL in these experiments revealed a distinct increase in NRL of ~4 bp from NT to iAs-T Beas-2B cells (from 191 to 196 bp - compare locations of the blue and red bars that indicate nucleosomal ladder in Figures 3A - B). Interestingly, our observed NRL changes correlate with the observed morphological changes in cells seen in Figure 2. The observed NRL changes are dose dependent (Figure 3C - D), an increase of ~5 bp is observed in HeLa cells treated with 0.5 μM iAs, with a further ~4 bp increase in 1 μM iAs-treated cells (166 bp in NT to 171 bp in 0.5 μM to 174.9 bp in 1 μM cells). Furthermore, removal of Na_3_AsO_3_ from 1 μM-transformed cells for 10 days (known as iAs-Rev) resulted in a ~3 bp decrease in the NRL (174.9 bp to 171.8 bp) (Figure 3C - D).

Our results suggest that iAs-induced cellular transformation results in chromatin with less periodicity and reduced average nucleosomal spacing, with the consequence being heterochromatinization. We further confirmed the increase in chromatin compaction by showing that chromatin from iAs-T cells was more resistant to stringent digest by MNase (Additional file 3: Figure S3). Decreased nucleosome periodicity and MNase inaccessibility are all typical of heterochromatin formation and transcriptional repression [23]. Our data showing increased NRL supports a role for arsenic in the assembly of repressive chromatin [5], although the mechanism is not clear.

**Figure 4:**
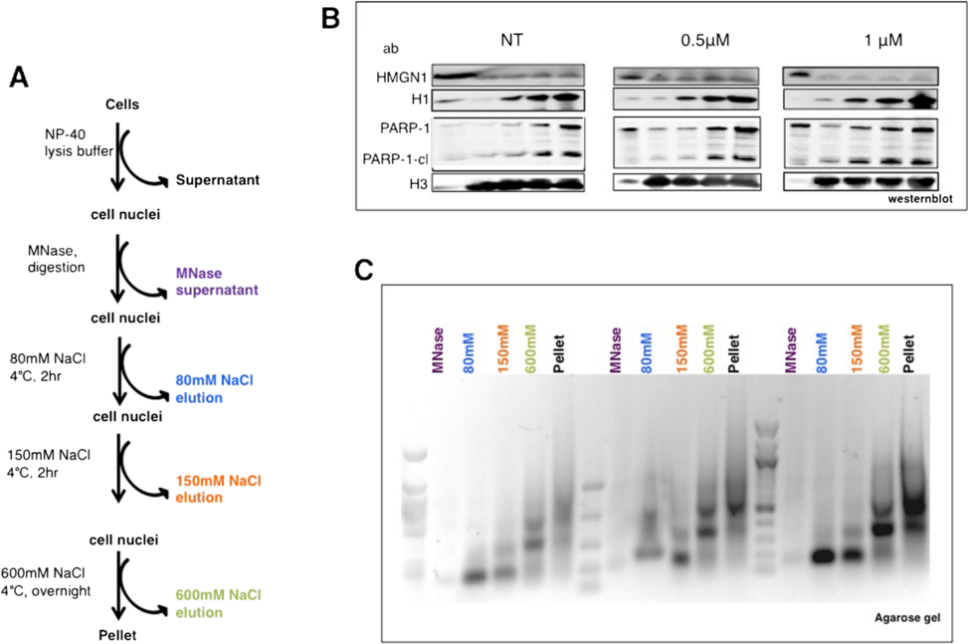
**Protein and DNA characterization of chromatin from HeLa cells with/without arsenic treatment. A)** Chromatin-fractionation procedure. **B)** SDS–polyacrylamide gel analysis (SDS-PAGE) of chromatin architectural proteins (CAPs) and other chromatin proteins in salt fractions. Equal aliquots of fractions from successive extraction steps in a typical experiment were loaded onto a 4-12% polyacrylamide gel, which was electrophoresed and westernblotted for the various proteins. **C)** Ethidium bromide–stained agarose gel showing a typical MNase ladder of DNA purified from MNase-treated nuclei (MNase); followed by ladders of DNA purified from the nuclear supernatant (Supn); successive 80 mM, 150 mM, and 600 mM extractions; and the remaining pellet, as indicated in **(A)**.

Furthermore we show arsenite treatment resulted in ~30% increase in histone H1 protein levels (data not shown), supporting our hypothesis of heterochromatization, as histone H1 is known to create heterochromatin and promote longer NRL. Histone H1 and other chromatin architectural proteins (CAPs), high mobility group protein-1 (HMGN1) and poly-ADP-ribose polymerase (PARP-1) bind reciprocally to create chromatin structures that specify transcriptional outcomes [23,25,26]. Therefore, we next tested whether this arsenic-induced upregulation of histone H1, changes CAP-chromatin association patterns. Consequently we carried out salt fractionation of chromatin (Figure 4A) from HeLa cells (NT, iAs-T with 0.5 μM and iAs-T with 1 μM Na_3_AsO_3_ for 45 days) respectively, with the expectation that different subsets of CAPs-bound chromatin would be detected in these three cell conditions. As shown by western blot analyses, though iAs treatment induced changes in the protein concentration of these CAPs it does not change the pattern of their association with specific chromatin fractions. For instance, though H1 concentration increased globally, its strong association with insoluble 600 mM NaCl and pellet chromatin fractions did not change. Likewise, though globally downregulated with iAs treatment, HMGN1 was mostly present in the soluble chromatin fraction (Figure 4B). There are likely two processes taking place with Poly-ADP-ribose polymerase (PARP-1). 1) Arsenite selectively interacts with the zinc finger domains of PARP-1 [27], and releases it from DNA. Our data supports this view as we see a dose-dependent increase in PARP-1 associated with the more soluble chromatin fractions. 2) We observe an increase in cleaved PARP-1, a marker of apoptosis, although an increase in apoptosis was not observed in the cells. We posit that that some amount of apoptosis is occurring, which was not detected by our bulk DNA fragmentation studies. Lastly, we detected a dose-dependent increase in insoluble chromatin as represented by the increase in the 600 mM and pellet chromatin fractions (Figure 4C). Overall, our findings of increased repressive H1-, decreased activating HMGN1- and PARP-1-bound chromatin, with a decrease in MNase accessibility, are consistent with increase heterochromatinization of chromatin by arsenite exposure.

### Profile of arsenic-mediated differentially expressed genes (DEGs)

After establishing that the observed phenotypic changes in iAs-T cells correlate with changes in chromatin structure, and considering that changes in chromatin structural dynamics typically result in alterations in gene expression, we sought to determine the genes whose expression is modulated in iAs-mediated EMT. Such analyses will likely identify the genes responsive to the long-term exposure to low dose of iAs (and iAs-transformation) on a genome-wide scale as well as their functional roles. For this analysis, RNA from HeLa NT cells and iAs-T HeLa cells were analyzed using the Affymetrix GeneChip® Human Transcriptome Array 2.0. First we filtered and retained differentially expressed genes (DEG) with an FDR < 0.05. Using SAM analyses, we identified 683 DEGs deregulated by iAs-T, with 270 under-expressed and 413 over-expressed (Additional file 4: Table S1). Given the low ratios of differential expression overall, we further narrowed our gene lists and considered a cut-off ratio of 1.2 fold as being potential biologically relevant. We first tested whether previously identified arsenic-altered genes were also identified in our study. We observed that the top downregulated genes include ion and anion transporters, as well as several zinc finger-binding proteins [28] (Additional file 5: Table S2). Additionally, some of the highly down-regulated genes observed include: Major Histocompatibility Complex, Class II, DR Alpha (HLA-DRA) cluster of differentiation 36 (CD36), collagen and homing cell adhesion molecule (CD44), while one of the most highly up-regulated genes in our studies is heme-oxygenase-1 (HMOX1) [29,30].

### Functional enrichment analyses of the arsenic-mediated differentially expressed genes

**Figure 5:**
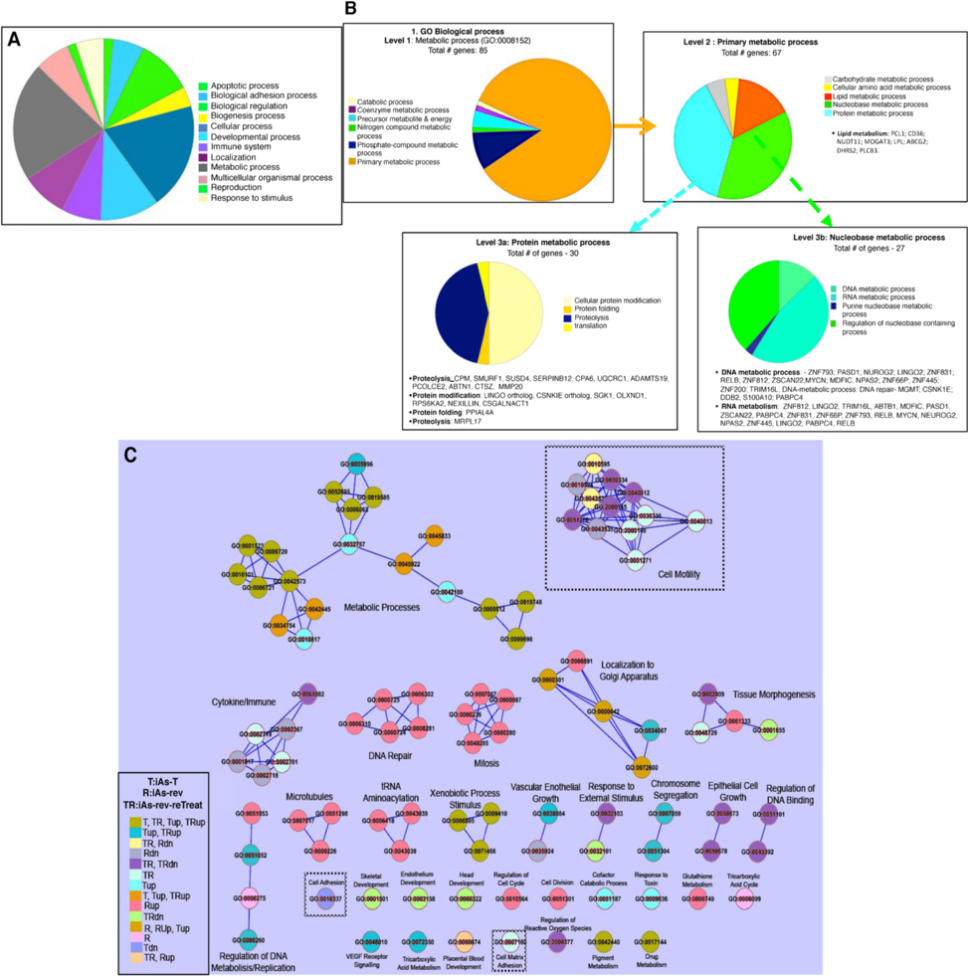
**Biological processes associated with arsenite-altered DEGs in cells. A)** Following PANTHER Pathway analysis, the biological processes linked to genes are illustrated. Each sector of the pie chart represents the total number of genes expressed for each cellular and biological function. Genes were filtered for an absolute value log2 ratio ≥1.2 and a significance value of p ≤ 0.05. **B)** In depth analyses of the metabolic processes of iAs-target genes. Level 1: Pie chart indicates relative proportions of genes found to be involved in this process. Level 2: Primary metabolic process and genes associated. Level 3a: genes involved in protein metabolic process while Level 3b: Genes involved in the nucleobase metabolic process. **C)** categoryCompare [34] showing the biological processes (grouped according to gene ontology terms) in which the DEGs (upregulated or downregulated) identified in each of the three conditions (iAs-T, iAs-Rev and iAs-rev-ReTreat) compared to NT. Each node represents a gene ontology (GO) term, and all of the nodes pose KEGG p-value < 0.05 and FDR q-value < 0.05. Color code is shown in the figure legend. Also indicated are processes that go either up or down in the various conditions. When no up or down is placed next to the condition, it means that some genes in this biological process that were up or downregulated. Shown also are significant terms including ‘metabolic processes’ and ‘cell adhesion’ in arsenic treatment (iAs-T and iAs-rev-reTreat), while Mitosis and DNA repair are among the most significant in iAs-rev cells. Tables used for creating this graph are in Additional file 7: Table S4.

We performed Protein ANalysis THrough Evolutionary Relationships (PANTHER) [31] on the DEGs, to identify significant gene ontology molecular function (GO MF) and gene ontology biological process (GO BP) terms. The purpose of this analysis was to find important biological functions and processes that characterize the impact of the DEGs identified in this study (Figure 5A & C). Among the GO BP terms identified by PANTHER, the most significantly enriched terms are “cellular process regulation”, and “metabolic process regulation” with 76 and 85 genes assigned to these terms respectively. And since heavy metals disrupt a wide variety of metabolic processes [32] we decided to analyze this group of genes further.

Metabolic processes are divided into ‘primary’ (required for cell growth and survival) and ‘secondary’ metabolic processes (not required in cell survival). Our analyses showed that arsenite exposure mainly targeted the “primary metabolic processes”. Proteins within this pathway are involved in carbohydrate, amino acid, nucleobase, lipid and protein metabolism (Figure 5B). Like other heavy metals, arsenic has been hypothesized to outcompete the binding of nutrient elements to regulatory proteins (receptors, transporters and storage proteins), resulting in marked aberrations in the metabolism of carbohydrate, protein/amino acids and lipids [32]. At the protein metabolic level, arsenic-mediated cellular transformation resulted in changes in the expression of genes involved in ubiquitination, lysosomal degradation, protein modification and proteolysis [2] and references therein. Consistent with this, we found several DEGs functioning in proteolysis, protein folding, protein phosphorylation and protein modification (Figure 5B), further confirming the role of iAs in these processes. At the nucleobase metabolic level (a primary metabolic process), iAs exposure mediates the expression of proteins involved in DNA repair processes, RNA metabolism and purine metabolic processes (Figure 5B). In addition, changes in the expression patterns of some transcription factors were observed. These results suggest that not only does arsenic selectively interact with zinc finger containing proteins and prevent their binding to DNA [33], but also that arsenite-mediated signaling pathways regulate the expression of certain transcription factors (Additional files 4 and 5: Tables S1 – S2 for iAs-mediated target genes and their corresponding transcription factors). Finally, we also present a visual summary of the arsenic mediated biological processes using ‘categoryCompare’ [34] (Figure 5C).

To further identify regulatory mechanisms that potentially underlie the arsenic-modulated transcript levels, we investigated whether binding sites for specific transcription factors were enriched computationally in the promoter regions of these iAs-modulated gene sets using Gene Set Enrichment Analysis (GSEA). These analyses identified an enrichment for the following transcription factors: E12 (p < 5.42e-07), FOXO4 (p < 5.94e-05), LEF1 (9.41e-05), Myc-associated protein z (MAZ with p < 0.0016), Nuclear factor of activated T-cells (NFAT with p < 0.0051), Forkhead RElated Activator 2 (FREAC2 with p < 0.00016), ETS2 (p < 0.0071), and GATA4 (p < 0.0051) amongst others (Additional file 6: Table S3). Since the expression of these transcription factors were not affected by iAs, we postulate that the iAs dependent modulation of chromatin structure results in differential binding of these transcription factors to their respective promoter target sites, with consequences in specific gene expression patterns. We hypothesize that this may be the mechanism by which iAs alters gene expression of key genes associated in cancer development. Indeed modulation of the expression of all of these transcription factors - E12 [35]; FREAC2 [36,37]; FOXO4 [38]; LEF1 [39]; NFAT [40]; GATA4 [41]; ETS2 [42] have been implicated in altered gene expression during EMT. Thus, rather than change the expression of these factors, iAs may modulate their function by mediating chromatin structures that disfavor functional binding to their target sites. However, further studies will be carried out to determine if this is true.

### Gene regulatory pathways modulated by iAs during the process of transformation

We next set out to identify the gene regulatory pathways that were activated in HeLa cells chronically exposed to a low dose of sodium arsenite producing EMT. We employed the GSEA to identify modulated KEGG (Kyoto Encyclopedia of Genes and Genomes) pathways in our microarray data (Additional file 7: Table S4). We did not observe changes in the gene expression levels of stress response genes, such as heat shock proteins in iAs-T cells. However the expression of some DNA repair proteins namely Endothelial pas domain 1 (EPAS1), CD36 and O-6-methylguanine-DNA methyltransferase (MGMT) were down regulated, while HMOX1 was upregulated. In addition, the PANTHER analysis revealed several altered pathways (Additional file 8: Table S5 respectively), including the ‘angiogenesis’, ‘Apoptosis’, ‘p53’, ‘Inflammation’, ‘Wnt signaling” and ‘Integrin signaling’ pathways being highly altered. These results suggest that iAs targets integrins to promote EMT. For disease associated pathways, there was an over-representation of cancer pathways and the genes found in the cancer modules are shown in Additional file 8: Table S5.

### DEG patterns after removal of Na_3_AsO_3_

In view of the observation that withdrawal of iAs, resulted in a ‘reversion’ both in cell morphology and NRL towards NT cells, we therefore sought to determine whether there was a concomitant alteration in gene expression. Hence, RNA from iAs-Rev cells (as shown in Figure 1) was analyzed using microarray analysis to profile the gene expression patterns. Comparison analysis of the DEGs between iAs-Rev and NT cells, found only 39 genes that were not differentially expressed in NT conditions. We theorize that because this set of genes did not revert to NT levels, that these genes are probably involved in the progression of the defunct gene expression states seen in iAs-T cells.

**Figure 6:**
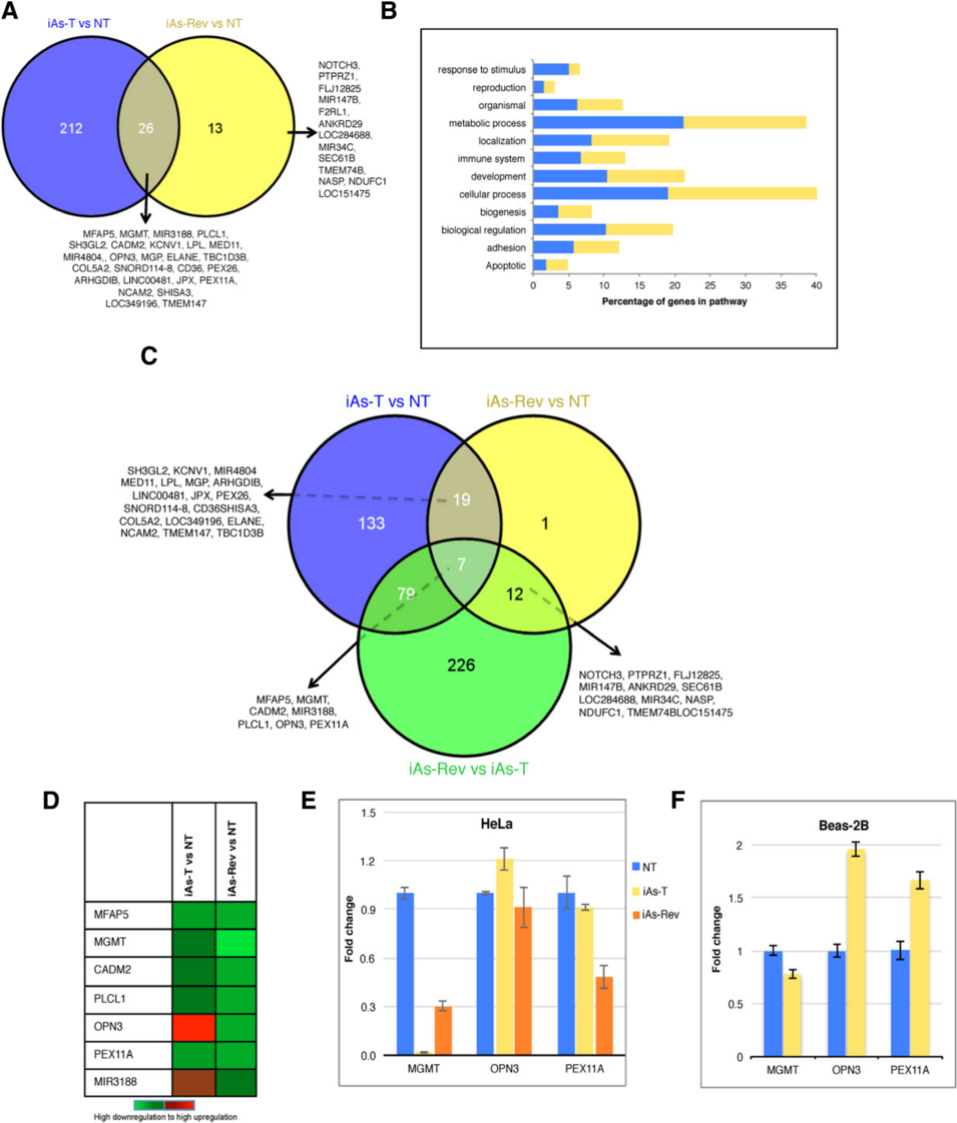
**Analyses of common genes and their biological function between iAs-T and iAs-Rev cells. A)** Venn diagram analyses showing the genes common between the two conditions. **B)** We used 100% stacked column analysis to compare the changes and biological functions of genes under arsenic treatment and in reversed conditions. Bar graph representing the percentage of genes and their biological process altered in iAs-T and after iAs is withdrawn from iAs-T, referred to as iAs-Rev cells. **C)** Identification of unique and shared genes between iAs-T and genes that did not revert after arsenic is withdrawn. The Venn diagram indicates the number of unique and common differentially expressed genes at each treatment condition. **D)** Heatmap analysis of gene expression changes from microarray data in iAs-T and iAs-Rev cells. The analysis shows relative fluorescence from green (downregulated) to red (upregulated) of the six genes found common in the experimental conditions. qRT-PCR analyses to validate the microarray data in **E)** HeLa cells and **F)** Beas-2B cells.

Next, we analyzed the changes in gene expression between iAs-T and iAs-Rev cells. Our results suggest that the exposure time period and the reversion time period had a significant number of common genes. Indeed, Venn-diagram analyses showed 26 DEGs common between these two cell populations (Figure 6A and Additional file 9: Figure S4). These data indicate that most genes that respond to iAs exposure and transformation reverted to their normal levels at 10 days after withdrawal of iAs. However, the expression of some key genes remain permanently altered, possibly resulting in the ability of these cells to differentiate and become tumorigenic. Further analysis of the different biological processes affected in iAs-T and iAs-Rev conditions revealed that the cellular and metabolic processes are the top-altered biological processes. Furthermore, withdrawal of iAs from iAs-T cells (iAs-Rev) resulted in the reduction of the percentile of genes within the ‘response to stimulus’ group, indicating that this process is no longer needed in the absence of the toxin. On the other hand, we observed an increase in the percentile of ‘apoptotic’ genes (Figure 6B), also correlating with the fact that in the absence of iAs, there is a decrease in the ability of these cells to transform. These results suppose that apoptotic resistance could be a mechanism for iAs-induced malignant transformation.

Finally, because we were primarily interested in identifying processes influenced during iAs-induced cellular transformation, we analyzed the DEGs found in the following conditions: NT vs. iAs-T, NT vs. iAs-Rev and iAs-T vs. iAs-Rev. Overlapping genes represent those genes that were permanently changed in iAs treatment and transformation (Figure 6C). Only seven genes were common in all three conditions: microfibrillar associated protein 5 (MFAP5)- associated with poor cancer prognosis [43-45], O-6-methylguanine methyl transferase (MGMT) - involved in the etiology of cancer [46], MIR3188 –important in post-translational modifications in cancers, cell adhesion molecule 2 (CADM2) a tumor suppressor [47], Phospholipase C-like 1 (PLCL1), Opsin 3 (OPN3), and Peroxisomal biogenesis factor 11 alpha (PEX11A). The MFAP5 level is decreased about 4.8 fold in iAs-T compared to NT cells. Interestingly, in iAs-Rev, its expression increased compared to iAs-T. However, its expression levels never returned to the levels in NT cells (Figure 6D - E). For MGMT, CADM2 and PEX11A, their levels went down in iAs-T cells, and remained low even in iAs-Rev cells. In the case of PCL1, compared to NT cells, its expression was downregulated in transformed cells and interestingly in reversed conditions the expression of PCL1 went up compared to NT cells. And lastly, the opposite was true for OPN3 and MIR3188, where the gene expression levels were up in iAs-T cells, but went down in iAs-Rev cells. Remarkably, some of these expression patterns were maintained in the same direction even when iAs was reintroduced into iAs-Rev cells (Additional file 10: Figure S5), suggesting a direct effect of iAs on the expression of these genes. These expression patterns were validated using qRT-PCR (qunatitative reverse transcription PCR) analysis for MGMT, OPN3 and PEX11A in HeLa cells. These changes in gene expression patterns were subsequently confirmed in BEAS-2B cells, indicating that these genes may universally be targets of iAs carcinogenesis (Figure 6F).

We next determined the biological relevance of these proteins, by using ‘STRING’ (Search Tool for the Retrieval of Interacting Genes/Proteins) [48] to examine their possible interacting partners. From the STRING gene/protein network analyses, it is clear that several molecular markers interacted strongly with the target genes/markers we studied here. For instance, MFAP5 interacts with several matrix factors, cell adhesion proteins and is predicted to inhibit NOTCH1 (Additional file 11: Figure S6A); MGMT interacts with several important DNA repair proteins, either directly or indirectly (Additional file 11: Figure S6B); OPN3’s interacting partners are collagen factors critical in the structural integrity of the cell (Additional file 11: Figure S6C); Pex11A on the other hand, interacts with PPARA, NCOA2, CREBBP, SMARCD3 (Additional file 11: Figure S6D) [12] while CADM2 interacts with several zinc finger transcription factors (Additional file 11: Figure S6E). Interestingly, most of these genes above have cell membrane functions and since dynamic changes of membrane structure are intrinsic to organelle morphogenesis and homeostasis, their disruption could be lethal. Lastly we used a systems biology tool, miRUPnet [49] to infer the functional importance of mir3188 and showed that most of its target genes are critical in several cancer pathways and its most significant ‘GO term’ is chromatin binding. Thus each of these proteins and microRNA could serve as important protein interaction/target hubs that if deregulated, will have important consequences in the normal development of a cell. Overall, we show dynamic changes in gene expression as some genes get reactivated in cells where iAs was introduced after reversal. In other cases very new genes that were earlier not activated became altered after reintroduction of iAs (iAs-rev-reTreat – cells) (Additional file 5: Table S2). These results imply that low doses of iAs trigger adaptive responses that alleviate the adverse effects of arsenite cytotoxicity and oxidative stress.

### Validation of the microarray data confirms the iAs-modulated chromatin structural changes mediate altered gene expression patterns

**Figure 7:**
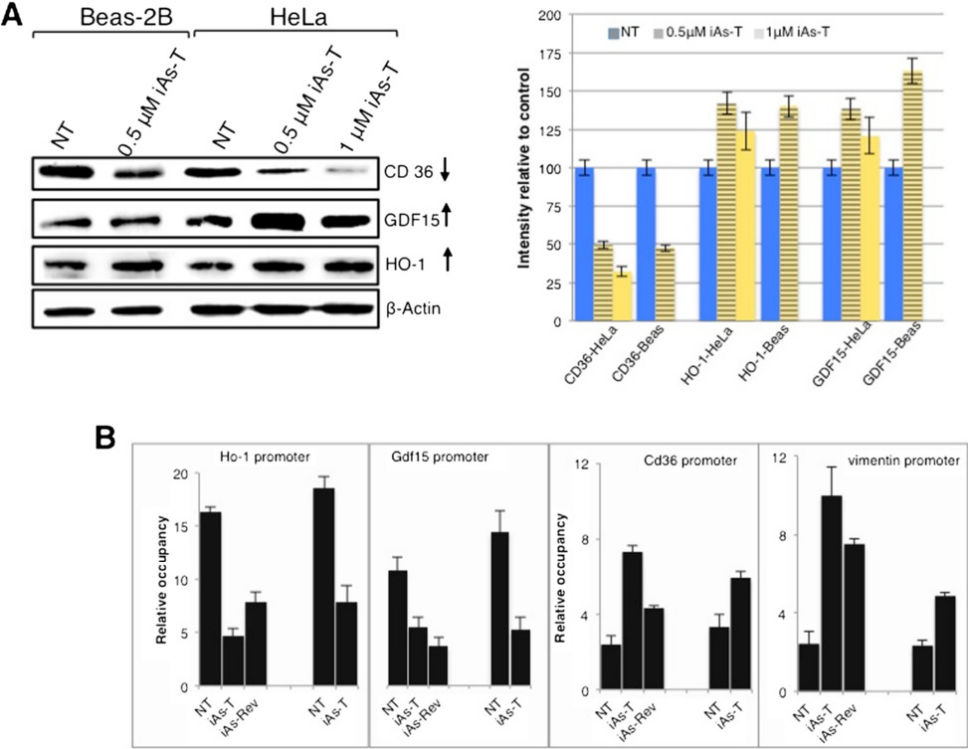
**Immunoblot analysis of iAs-target genes in both HeLa and BEAS-2B cells. A)** Shown are representative immunoblots with expanded views of antibody-decorated bands. Immuno-decoration was carried out with primary antibodies against the respective proteins CD36, GDF15, HO-1. Besides the individual immunoblots are shown panels, which give a graphical representation of the immuno-decoration levels of the various proteins (Student’s *t*-test p < 0.05). **B)** MNase-treated DNA from cells from all experimental conditions was subjected to quantitaitive real-time PCR analysis using primers that map to the promoters of the respective genes. Note that changes in nucleosome occupancy correlates with the gene expression patterns of the various genes in the different experimental conditions. The average results ± SD from two independent experiments are shown.

We used both Western blot analyses as well as qRT-PCR to confirm changes in gene expression both at the protein and transcript levels respectively. Selected from the iAs-modulated networks, Western blot analyses confirmed the upregulation of HMOX1 and growth differentiation factor 1 (GDF1) in iAs-T cells, and downregulation of CD36 and claudin3 (CLN3) (Figure 7A and Additional file 1: Figure S1). Additionally qRT-PCR expression validated some of the targets shown earlier and the values did not differ significantly from microarray values (p > 0.05) (Figure 6D). Furthermore, we show that these changes in gene expression pattern correlate with iAs-mediated changes in nucleosome occupancy over the promoter regions of these genes (Figure 7B). These results further support our idea that iAs-induced changes in chromatin structural rearrangements are coupled with changes to gene expression patterns.

### IAs-exposure mediates alternative splicing of specific genes

**Figure 8:**
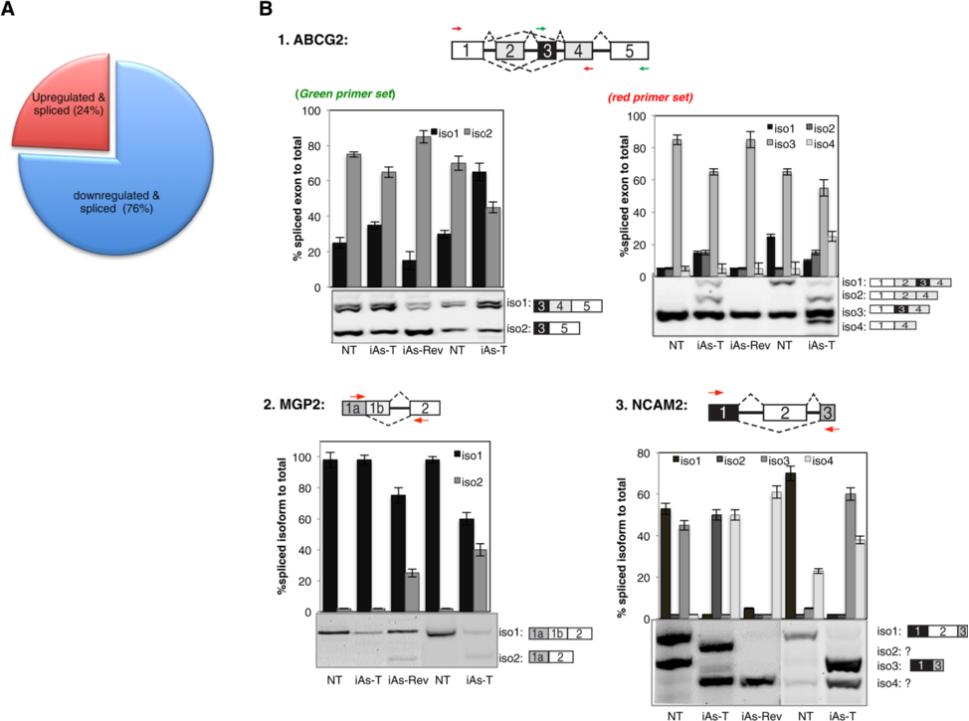
**Splicing-sensitive human microarray analysis. A)** Distribution of genes that change in gene expression and AS in iAs-T conditions. Genes whose expression is upregulated and show changes in ASEs (red); genes whose expression is downregulated and show changes in ASEs (blue). **B)** Examples of RT-PCR analysis of iAs-mediated alternative splicing events are shown at three genes: ABCG2, MGP, and NCAM2. Primer locations for each RT-PCR assay are shown by arrows. Each experiment was repeated at least thrice, and data are mean ± SEM with p < 0.05 (student’s *t* test). The list of iAs-regulated alternative splicing events and the primers used to validate microarray data are available upon request.

Organisms use alternative splicing to increase transcriptome diversity necessary to cope with environmental stress. We therefore asked whether iAs effects specific alternative splicing isoforms needed for the adaptive behavior of cells treated with a low dose of iAs. We used the GeneChip® Human Transcriptome Array 2.0 (HTA 2.0) for analyzing differential gene expression studies to analyze differential splicing patterns in NT and iAs-T cells. Interestingly, iAs was found to induce alternative splicing events in 104 genes (p < 0.05) (Additional file 12: Table S6), indicating that iAs does not impact the general splicing mechanism but is specific for a subset of genes. Interestingly ~75% of these alternative splicing events occur in genes whose expression levels were downregulated during iAs-transformation (Figure 8A). We therefore asked whether the observed splicing patterns were due to down regulation of the genes rather than splicing. To answer this question, we analyzed splicing patterns of two downregulated genes - Matrix gla protein 1 (MGP1) and Neural Cell adhesion molecule 1 (NCAM2) and an upregulated gene - ATP-binding cassette sub-family G member 2 (ABCG2) [50]. Semi-quantitative RT-PCR was performed on total RNA samples harvested from NT, iAs-T and iAs-Rev HeLa cells; and NT and iAs-T BEAS-2B cells, using primers designed to distinguish between the variants. Results of the changes in specific isoform expression patterns are shown in Figure 8. In the case of ABCG2, in iAs-T cells (HeLa as well as BEAS-2B), there was an increase in expression of this gene. Furthermore, exposure to iAs, resulted in the expression of specific isoforms (Figure 8B). For the MGP gene, generally there was downregulation of the expression of this gene transcript confirming the microarray data. Interestingly, there was an increase in the expression of isoform 2 in iAs-rev HeLa cells while the increase in this isoform occurred in iAs-T BEAS-2B cells. We speculate that these differences might be due to cell-type specificity or the degree of tumorigenesis (bearing in mind that HeLA cells are carcinogenic) (Figure 8B). However, the results for NCAM2 were even more dramatic. Two bands were observed in NT cells, both representing known isoforms. Strikingly, the longest isoform with all three exons is absent in iAs-T and iAs-Rev HeLa cells. The next known isoform, isoform 3 having exons 1 and 3, was again absent in all other conditions except in iAs-T-BEAS-2B cells. While these known isoforms were observed, bands representing putative novel isoforms (isoforms 2 and 4) were seen in iAs-T HeLa cells, and only isoform 4 was seen in iAs-Rev HeLa cells. In BEAS-2B cells, some level of expression of isoform 4 was seen in NT cells, and the amount of it increased significantly in iAs-T BEAS-2B cells. It is possible that these isoforms are needed for cell-type specificity (difference between NT BEAS-2B and HeLa) as well as in cellular adaptation to arsenic exposure (appearance of isoform 4 in iAs-T BEAS-2B and HeLa cells). More studies are therefore required to delineate the relevance of each of these NCAM2 isoforms in iAs-induced carcinogenesis. Taken together, these results demonstrate that iAs can impact alternative splicing of a subset of genes, critical in disease pathology. To date, there have been no studies implicating arsenic in the regulation of splicing or the splicing machinery and it will be interesting for future studies to determine how each of these isoforms potentiate the carcinogenic po-tential of iAs.

## Discussion

iAs is a well-established carcinogen that induces a number of pathological diseases including several cancers [3-5]. However, the molecular mechanism underlying As^3+^ induced disease pathology and the downstream genes that mediate As^3+^ carcinogenicity are not completely understood. Several studies have been carried out to determine genes as well as pathways involved in arsenic-induced cellular adaptation to toxicity and pathogenesis [3]. Adaptation includes alterations of genes that are needed for cellular survival in their new environment. Thus the identification of iAs-induced gene alterations at the transcriptional and post-transcriptional levels is required to fully understand As^3+^ mediated cellular adaption, and to date has been lacking. To fill this gap we have carried out a systematic and comprehensive study to determine the structural changes to chromatin and cellular changes elicited by arsenic exposure.

First we show that chronic exposure of ‘normal’ human bronchial epithelial BEAS-2B cells to low doses of iAs significantly enhanced their ability to grow and undergo EMT. Likewise, the immortalized carcinoma HeLa cells underwent further EMT after chronic exposure to low-dose iAs. This is in line with previous studies showing that HeLa cells can undergo EMT [51-53]. Second, we show that in both cell types, iAs-T cells had increased nucleosome repeat length, indicative of heterochromatinization. Correspondingly the levels of chromatin-bound histone H1 increased with a concomitant decrease in the activating CAP HMGN1. These changes in chromatin structure correlate with alterations in gene regulation both with respect to transcription initiation and alternative splicing. Third, the gene expression profile of iAs-Rev cells showed a remarkable reversal of many chromatin and gene expression patterns. However, some key genes that might be oncogenic remain altered in these reversed conditions. Interestingly, iAs-induced chromatin changes facilitate the altered gene expression patterns (Figure 5). Fourth, not only are gene expression patterns altered but microRNAs expression is altered as well, suggestive of their function in iAs-induced pathogenesis (Additional file 13: Table S7). Some of these miRNAs regulate the expression of known oncogenes or tumor suppressor genes, thus acting as onco-miRNAs or tumor-suppressor miRNAs [54]. Fifth, we show for the first time, that arsenic exposure results in both changes in gene expression and in specific transcript isoform expression, possibly necessary for the adaptive property of these cells (Figure 8). Together these data indicate that altered gene expression is a major consequence of chronic As^3+^ exposure.

Thus our data unveils known and novel pathways involved in iAs-EMT and suggests that iAs activates several signal transduction cascades that lead to changes in chromatin structure. Bulk chromatin analyses of iAs-T cells show an increase in NRL indicative of heterochromatinization and withdrawal of iAs as in iAs-rev, results in reduced heterochromatinization. Further supporting heterochromatinization, is our observation of increase in histone H1-chromatin binding and upregulation of DOT1L, the only known H3K79me3 methyltransferase [55] in iAs-T cells (Figures 3 – 4). These results are in accordance with previous studies reporting arsenite-induced increase and decrease in repressive and activating histone marks respectively [56]. In addition, recent epidemiological studies confirm that iAs significantly increases DNA hypermethylation in a dose-responsive manner at the promoters of oncogenes [56,57]. Although our studies do not directly measure changes in histone PTMs, we did detect changes nucleosome occupancy at several iAs-target genes. These studies hint at a potential mechanism by which iAs-mediated effects alter chromatin structure resulting in positive or negative effects on gene regulation.

We also show that specific changes in gene expression accompany arsenic treatment and withdrawal (Figure 4). The consequences of repeated and constant arsenite exposure to cells are manifested by the development of tolerance, accompanied by changes in chromatin structure and gene expression patterns. Since humans are frequently on the move, relocation to a newer environment without the constant insult from arsenite will result in the establishment of new gene expression patterns. However, the possibility of developing cancer still exists based on gene expression patterns that remain altered even when iAs insult is discontinued (Figure 7C). Indeed we show in each of the experimental conditions: iAs-T, iAs-Rev and iAs-rev-reTreat, that cells adapted by changing the expression of genes both at the transcription and splicing levels. We observed changes in the expression of specific microRNAs, (Additional file 13: Table S7) suggesting a role of these microRNAs in the adaptive responses to arsenic exposure and pathogenesis. It is possible that the change in gene expression of specific microRNAs is a mechanism through which iAs exposure regulates the levels of key proteins. One of these microRNAs modulated by iAs, miR124-1, was recently shown to target Slug to regulate EMT and metastasis [58]. Overexpression of miR200b has been implicated in the reversal and prevention of arsenic-induced malignant transformations in lung cells [59]. These studies highlight the importance of microRNAs in targeting specific proteins and driving specific cellular pathways, especially cancer pathways [58-62]. Four out of the seven significantly altered microRNAs target genes important in cancer pathways, implicating the deregulation of these microRNAs in arsenic induced carcinogenesis.

Chromatin structure regulates both transcription initiation and co-transcriptional splicing [63,64]. Therefore it is likely that in modulating chromatin structure, arsenic directly affects splicing patterns and/or indirectly by modulating the expression of splicing factors (Additional file 12: Table S6). Arsenic has been reported to affect both positive [65] and negative [66] alternative splicing events. Organisms use alternative splicing mechanisms to enhance their ability to cope with stress via transcriptome plasticity [67]. Conceivably, iAs-induced alteration of gene splicing patterns may underlie the mechanism of iAs-induced disease pathology. While our exon array and the validation analyses focused on finding alternative splicing events that were present in our study, we also found evidence for considerable heterogeneity. For example, some of the NCAM2 splice variants differed in response to the various treatments, suggestive that some of these NCAM2 isoforms may be potentiating the metastatic potential of arsenic.

## Conclusion

Overall, our comprehensive genome-wide study provides new insights into markers and mediators of arsenite responses within a cell. It also identifies known and novel regulatory pathways involved in the toxicological action of arsenite. Such detailed and comprehensive studies are important in dissecting the cause and effect of iAs exposure on signal transduction pathways and its consequences to gene regulatory mechanisms. While iAs is involved in carcinogenesis, it is also used in the treatment of acute promyelocytic leukemia (APL) [68]. It is possible that the anti-carcinogenic and carcinogenic actions of arsenite share a common molecular intersection that is related to level of arsenite exposure (high dose vs. low dose), length of exposure (e.g. chronic vs. acute), and/or exposure to the arsenic species (e.g. arsenite, arsenate, MMA, DMA). Thus, it will be important to ask whether epigenetic changes also mediate arsenic-cancer therapy. Our studies therefore provide a platform to begin to define these epigenetic changes.

## Methods

### BEAS-2B and HeLa cell culture

Cells were obtained from ATCC and cultured maintained in DMEM supplemented with 10% FBS and 1% penicillin and streptomycin in a humidified atmosphere with 5% CO_2_ at 37°C. Cells were passaged regularly and subcultured to ~80% confluence before conducting the experimental procedures.

### Antibodies

The antibodies cd36, vimentin, claudin3, GDF, β-actin and HO1 were obtained from Abcam® while PARP1, HMGN1 and H1 were obtained from Activemotif®.

### Population doubling number (PDN)

To determine the population doubling number, 4 × 10^6^ cells were plated in 3 cm plates. After 24 hrs the medium was removed and exchanged for culture medium containing 0.5 μM or 1 μM of Na_3_AsO_3_ (Sigma-Aldrich) and incubated for 5, 10, 15, 21 days. After the treatment period the cells were washed with PBS and harvested using trypsin/EDTA. The cells were then counted and the population doubling numbers were calculated using the equation the population doubling number = (logN/N_0_ × 3.31) where N is the number of cells at the end of the culture period and the N_0_ is the number of the cells plated.

### Cell transformation by arsenite exposure

BEAS-2B and HeLa cells were continuously exposed to vehicle control (deionized H_2_O) or 0.5 μM or 1 μM of arsenite (Na_3_AsO_3_, Sigma-Aldrich), respectively. When reaching about 80–90% confluence, cells were sub-cultured and Na_3_AsO_3_ was then added to cells after overnight attachment. These procedures were repeated every 3 or 4 days for 16 weeks. During the exposure period, cell morphology changes were monitored. Cell malignant transformation was assessed by changes in cell morphology and EMT marker protein levels.

### Cell growth assays

All media were purchased fresh and appropriate amounts of supplements were added as indicated by the manufacturer. Cells were incubated at 37°C with humidified air and 5% CO_2_. Cells were harvested after each culture ensuring that the cells had ~95% cell viability. Harvested cells were centrifuged for five minutes at 200-x g. Samples were taken each day for counting. Cell viability was determined by trypan blue dye exclusion using a hemocytometer and calculated as percent viability times total cells/ml.

### Nucleosome-repeat length analyses

Nucleosome-repeat length analysis was done according to Nalabothulla *et al* [23].

### Salt fractionation of chromatin

Salt fractionation of chromatin was done according to Teves *et al* [69].

### DNA laddering analysis for apoptosis

DNA fragmentation analysis (DNA ladder) was assessed by agarose gel electrophoresis according to [22,70] with a slight modification. iAs-treated or NT HeLa and BEAS-2B (2 ×10^6^ cells) were collected and centrifuged at 1200 rpm for 5 min and then re-suspended in a lysis buffer [50 mM Tris-HCl pH 8.0, 5 mM ethylenediamine tetraacetic acid (EDTA), 1.2% sodium dodecyl sulfate, 150 mM NaCl, 0.2 mg per ml proteinase K] followed by incubation at 37°C overnight. Cellular DNA was isolated by phenol extraction and the DNA samples were carefully loaded into the wells of a 2.0% agarose gel. Electrophoresis was carried out in TAE buffer at 50 V for 1 h and the DNA was visualized by ethidium bromide staining.

### RNA extraction and Array hybridization

Total RNA was isolated from cells using a miRNeasy mini kit (Qiagen) and quality assessment was conducted using RNA 6000 Nano-labchip (Bioanalyzer, Agilent) and quantified by a Nanodrop spectrophotometer (Thermo). For transcriptome assay, total cellular RNA (100 ng) was processed to generate labeled cDNA following the Affymetrix protocols. The yield of labeled cDNA ranged from 6.27 ug to 7.57 ug among the 8 samples, of which 4.7 ug cDNA was applied to Affymetrix Human Transcriptome 2.0ST arrays (HTA2) for hybridization, one RNA sample per array. The labeling of RNA samples and hybridization of HTA2 arrays were performed at the University of Kentucky microarray core facility. The benefit of this array is to highlight spliced RNA isoforms using both exon and exon-exon junction probes that can measure excluded or included exons/regions. HTA 2.0 ST arrays were scanned using the Affymetrix 3000 7G scanner and the signal intensity of probe hybridization was processed using Command Console software version 4.1.2.

#### Gene level analyses

The initial gene expression patterns were done as follows: Signal intensities of the scanned arrays (.CEL) of all 8 samples were imported into Partek Genomics Suite 6.6 (Partek, MO) using GCRMA algorithm. Array exon probes were assembled into genes for statistical analysis at gene-level to assess significant differential expression using 1-ANOVA, followed by post-hoc paired comparisons among the 4 treatment conditions. More detailed analyses were done as follows: Analysis at the gene level first required probe set summarization and normalization. The raw ‘CEL’ files were processed using the Affymetrix® Expression Console Software (build 1.3.1.187) with “Gene Level - Default: RMA-Sketch” normalization to produce ‘CHP’ files for each of the eight samples. The eight CHP files were then imported into the Affymetrix® Transcriptome Analysis Console (TAC) 2.0 (build 2.0.0.9), using the “Gene Level Differential Analysis” option. Four conditions were created within TAC: NT, iAs-T, iAs-Rev, and iAs-Rev-T according to the conditions in Fig. I. For each condition, the two corresponding replicates were added. Next, the “Run Analysis” step was performed to determine differentially expressed transcript clusters (DETCs). Note that transcript clusters have been annotated according to Affymetrix®, and similar to probe sets, do not have a one-to-one correspondence with protein coding genes. Many of the transcript clusters correspond to non-coding RNAs (lincRNAs, snoRNAs, miRNAs) while others have a many-to-one relationship between transcript clusters and genes. Comparisons were made to determine DETCs relative to the NT. An ANOVA *p-value* cutoff of 0.05 was used for each comparison, along with a log_2_ fold change of ±1.2 (as determined by Tukey’s bi-weight average). The use of p-value cutoffs alone in microarray gene expression studies leads to the potential underestimation of variance, which can result in a large number of false positive differentially expressed genes. Therefore, we additionally incorporated a fold change (FC) cutoff to help reduce the false discovery rate (FDR). We also used volcano plots to demonstrate the relationship between p-values and fold-change cutoffs. These plots are used in cases where use of only the p-value or the fold-change alone can lead to results that are not reproducible, particularly in the case of genes with low expression levels. Our choice of a log_2_ FC cutoff of 1.2 (FC of 2.3 up or down regulated) reduces the set to a manageable size, resulting in meaningful interpretation with a reduced FDR of 0.05. This sort of selection criterion is consistent with the results of the MicroArray Quality Control (MAQC) project [71] which determined that the use of FC criterion enhances reproducibility, and P-value criterion balances sensitivity and specificity.

### RNA transcript alternative splicing analysis

Alternatively spliced variants of RNA transcripts were analyzed using the Partek Genomics suite 6.6 software (Partek, MO). Briefly, HTA2 array data file (.CEL) of all 8 samples were imported into Partek GS and the microarray data was normalized using GCRMA algorithm. Exon probes were summarized into genes and then, alt-splicing ANOVA-1-way among treatment groups was run at the gene-level. Gene transcripts were identified as statistical significant for alternative splicing at p-value < 0.05.

### Categorical annotation

Once differentially regulated genes and exons were determined, a number of different analyses were used for functional enrichment including: PANTHER [31,72] and KEGG [73], which finds statistically overrepresented GO terms within the provided data set; categoryCompare [34] which provides a cross-platform and cross-sample comparison of high-throughput data at the annotation level. Furthermore, STRING [48] was used to assess protein-protein interactions. Finally, gene lists based on disease status were analyzed by GSEA [74].

### Gene expression and quantitative reverse transcriptase PCR

Total RNA was isolated using Zymoresearch Quick-RNA™ MiniPrep kit according to the manufacturer’s extraction protocol (R1054). cDNA was generated from 1μg of total RNA using the Superscript III First-Strand Synthesis System (Life Technologies). Analysis of mRNA was then accomplished using primers specific to each of the target mRNAs. RT-qPCR reactions were performed using EvaGreen® (Biotium) and Biorad CF96 following the manufacturer’s instructions and the resulting Ct values were normalized to *GAPDH*. Primers for microarray data validation are available upon request.

### Splice variant analysis in the validation series

The validation set was used to measure the predicted splice variants of 8 genes using specific primers (available upon request). 1.3 ng of cDNA were analyzed in duplicate to quantify spliced and unspliced forms by qRT-PCR. Results were run on an agarose gel stained with GelStar™ Nucleic acid Gel stain (Lonza) and analyzed on a typhoon for semi-quantitative analyses.

### Accession numbers

Data analyzed have been deposited in GEO with accession numbers GSE60760.

## Additional files

Additional file 1: Figure S1. iAs modulates the expression of key EMT genes in a dose-dependent manner (Figures 1D and 7A). Protein levels from 3 gels were normalized to the expression of β-actin in both BEAS-2B and HeLa cells. Data are mean S.E.M. of 3 independent experiments; *P* < 0.01. Additional file 2: Figure S2. Low dose of sodium arsenite does not induce DNA fragmentation in A) BEAS-2B cells and B) HeLa cells. DNA from control non-exposed cells and arsenic exposed cells were purified (see Figure 2) and ran on a 3.3% Nusieve agarose gel electrophoresis. Number of days in culture is shown in figure. NT: non-treated or control cells. T: iAs treated cells. High concentration of iAs (100 μM) shows the typical DNA fragmentation pattern (as indicated by arrows). Additional file 3: Figure S3. Arsenite treatment and transformation resulted in cells with more compact chromatin. Equal amount of nuclei was used and digest of chromatin from NT, iAs-T with 0.5 μM and 1μM iAs respectively cells showed more resistance to micrococcal nuclease (MNase). Interestingly removal of iAs from 1μM iAs-T, showed an increase in chromatin accessibility to MNase. Chromatin accessibility is dose-dependent as more resistance to MNase is seen in chromatin from cells transformed with 0.5 μM iAs compared to 1 μM iAs. Additionally, increase in accessibility is observed in cells from which iAs is removed (iAs-rev). Additional file 4: Table S1. Genes upregulated and downregulated by iAs exposure. Additional file 5: Table S2. Heatmap showing the differential gene expression patterns of different solute carrier proteins and zinc-finger binding proteins modulated in the different experimental conditions. Additional file 6: Table S3. Transcription factors whose binding sites were detected at the promoters of iAs-target genes. Additional file 7: Table S4. Pathways involved by the genes targeted by iAs. Detailed analyses of the iAs-targeted genes and their association with cancer (analyzed using GSEA). Additional file 8: Table S5. Genes altered by iAs and their association with various cancer modules. Additional file 9: Figure S4. Genes common in both iAs-T and iAs-Rev cells. These analyses show genes that did not revert to NT conditions. Also shown are the functions of some of these genes. Additional file 10: Figure S5. Heatmap of the expression pattern of genes common in iAs-T, iAs-rev and iAs-rev-reTreat cells. Red: indicates upregulation and green indicates down regulation. From dark green to light green = low fold change downregulation to more down regulation. Dark red to light red = degree of upregulation: small to high upregulation. Additional file 11: Figure S6. Protein interaction map of altered *iAs*-target genes. The bioinformatics STRING database (version 9.1) was used to generate a protein interaction map with known and predicted protein associations that include direct physical and indirect functional protein linkages of microarray identified iAs-target genes at protein level from Additional file 10: Figure S5). Shown also are the interactors based on evidence (left side) and confidence level (right side). i) Evidence view: different line colors represent the types of evidence for the association. Green: neighborhood; red: gene fusion; blue: co-occurrence; black/grey: co-expression; pink: experiments; teal: databases; Pea green: textmining; purple: homology ii) Confidence view: Thicker lines represent stronger associations. Additional file 12: Table S6. Genes with iAs-mediated alternatively spliced events. Additional file 13: Table S7. MicroRNAs altered during arsenite exposure.
