# Supplementary figures and images for: Inorganic Arsenic-induced cellular transformation is coupled with genome wide changes in chromatin structure, transcriptome and splicing patterns
Source: BMC Genomics. 2015 Mar 19;16(1):212. doi: 10.1186/s12864-015-1295-9 (PMC4371809; doi:10.1186/s12864-015-1295-9)

Additional File 1: Figure S1

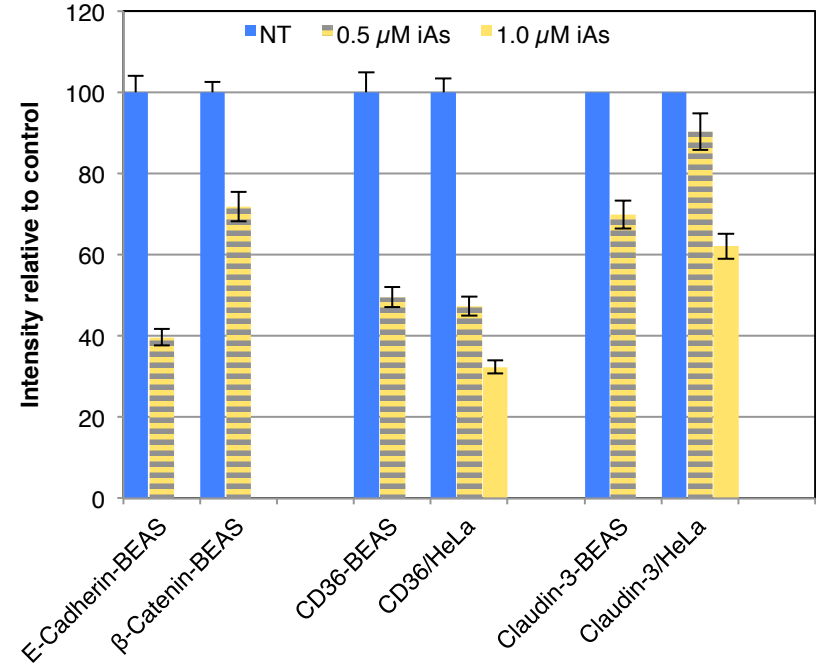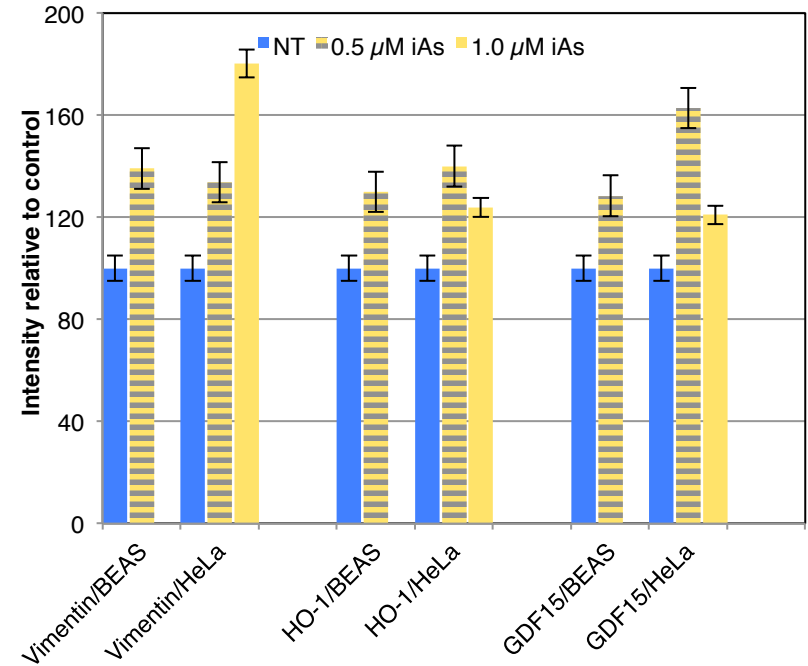

Supplement: Additional file 1: Figure S1. — iAs modulates the expression of key EMT genes in a dose-dependent manner (Figures 1D and 7A). Protein levels from 3 gels were normalized to the expression of β-actin in both BEAS-2B and HeLa cells. Data are mean S.E.M. of 3 independent experiments; P < 0.01. [file 12864_2015_1295_MOESM1_ESM.pdf]
