# Supplementary material for: Inorganic Arsenic-induced cellular transformation is coupled with genome wide changes in chromatin structure, transcriptome and splicing patterns
Source: BMC Genomics. 2015 Mar 19;16(1):212. doi: 10.1186/s12864-015-1295-9 (PMC4371809; doi:10.1186/s12864-015-1295-9)
Supplement: Additional file 2: Figure S2. — Low dose of sodium arsenite does not induce DNA fragmentation in A) BEAS-2B cells and B) HeLa cells. DNA from control non-exposed cells and arsenic exposed cells were purified (see Figure 2) and ran on a 3.3% Nusieve agarose gel electrophoresis. Number of days in culture is shown in figure. NT: non-treated or control cells. T: iAs treated cells. High concentration of iAs (100 μM) shows the typical DNA fragmentation pattern (as indicated by arrows). [file 12864_2015_1295_MOESM2_ESM.pdf]

Additional File 2: Figure S2

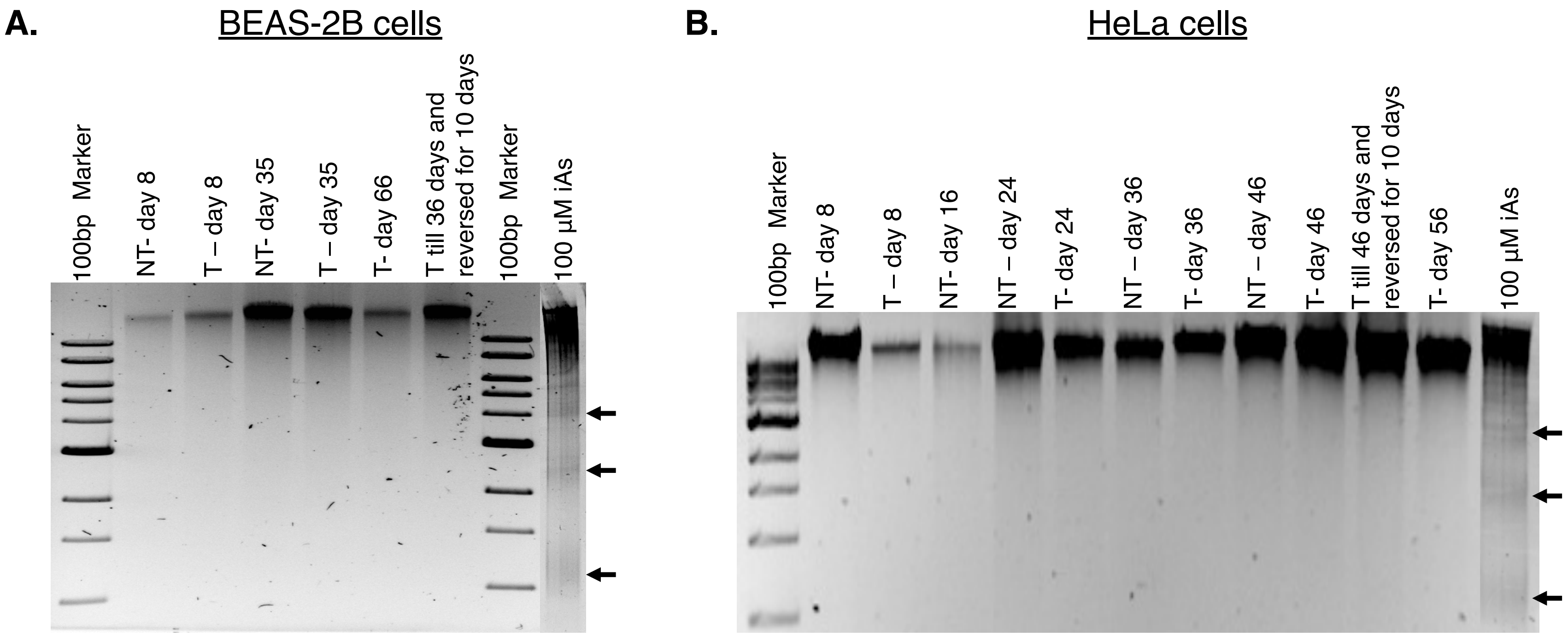

Additional File 2: Figure S2: Low dose of sodium arsenite does not induce DNA fragmentation in A) BEAS-2B cells and B) HeLa cells. DNA from control non-exposed cells and arsenic exposed cells were purified (see Figure 2) and ran on a 3.3 % Nusieve agarose gel electrophoresis. Number of days in culture is shown in figure. NT: non-treated or control cells. T: iAs treated cells. High concentration of iAs (100 μM) shows the typical DNA fragmentation pattern (as indicated by arrows).
