# Supplementary material for: Inorganic Arsenic-induced cellular transformation is coupled with genome wide changes in chromatin structure, transcriptome and splicing patterns
Source: BMC Genomics. 2015 Mar 19;16(1):212. doi: 10.1186/s12864-015-1295-9 (PMC4371809; doi:10.1186/s12864-015-1295-9)
Supplement: Additional file 3: Figure S3. — Arsenite treatment and transformation resulted in cells with more compact chromatin. Equal amount of nuclei was used and digest of chromatin from NT, iAs-T with 0.5 μM and 1μM iAs respectively cells showed more resistance to micrococcal nuclease (MNase). Interestingly removal of iAs from 1μM iAs-T, showed an increase in chromatin accessibility to MNase. Chromatin accessibility is dose-dependent as more resistance to MNase is seen in chromatin from cells transformed with 0.5 μM iAs compared to 1 μM iAs. Additionally, increase in accessibility is observed in cells from which iAs is removed (iAs-rev). [file 12864_2015_1295_MOESM3_ESM.pdf]

Additional File 3: Figure S3

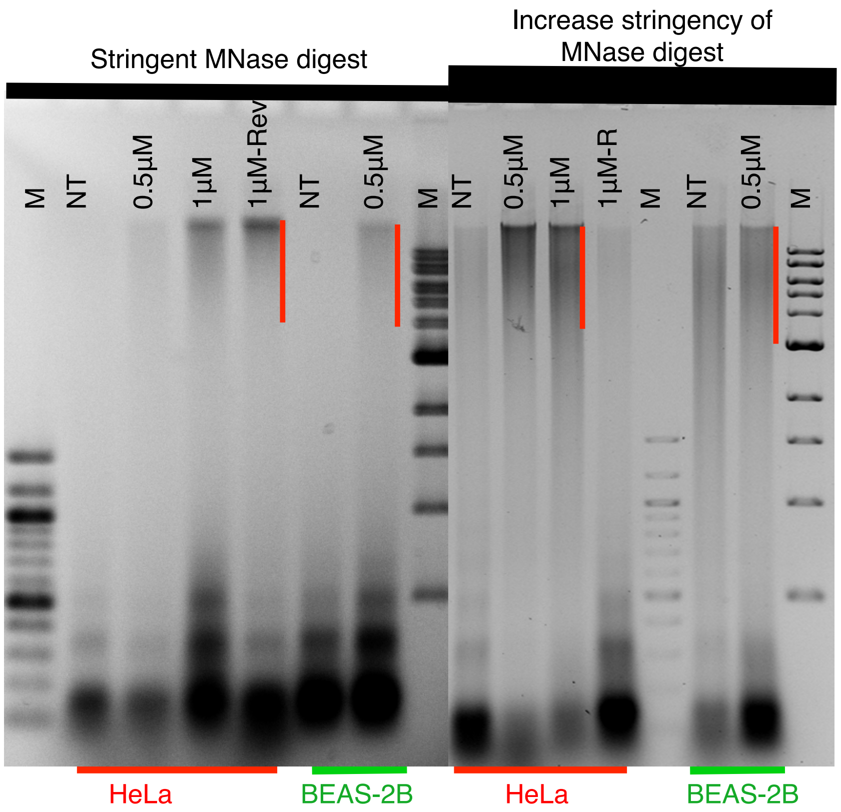

Additional File 3: Figure S3: Arsenite treatment and transformation resulted in cells with more compact chromatin. Equal amount of nuclei was used and digest of chromatin from NT, iAs-T with 0.5 and 1µM iAs respectively cells showed more resistance to micrococcal nuclease (MNase). Interestingly removal of iAs from 1µM iAs-T, showed an increase in chromatin accessibility to MNase. Chromatin accessibility is dose-dependent as more resistance to MNase is seen in chromatin transformed with 0.5µM iAs compared to 1µM iAs. Additionally, increase in accessibility is observed in cells from which iAs is removed (iAs-rev).
