# Supplementary material for: Inorganic Arsenic-induced cellular transformation is coupled with genome wide changes in chromatin structure, transcriptome and splicing patterns
Source: BMC Genomics. 2015 Mar 19;16(1):212. doi: 10.1186/s12864-015-1295-9 (PMC4371809; doi:10.1186/s12864-015-1295-9)
Supplement: Additional file 4: Table S1. — Genes upregulated and downregulated by iAs exposure. [file 12864_2015_1295_MOESM4_ESM.pdf]

Additional File 4: Table S1: Genes modulated by iAs

Genes downregulated at fold of >1.2 and FDR<0.05

UCA1	ZNF735	TTLL1	HACL1	KCNC4
ZNF831	NPAS2	ITGA6	TSPAN18	LPHN1
MFAP5	OR5V1	LRRC6	RNU7-63P	ATG10-AS1
ARHGDIB	EDNRA	HLA-B	CSNK1E	PTP4A3
MGP	L1CAM	SIDT1	ENTPD5	HLA-H
CD36	ETV4	RIBC2	AHSP	HLA-F
HLA-DRA	CTSZ	TUBB1	CHSY3	HLA-F
HLA-DRA	RIMKLBP1	RHPN2	DPAGT1	HLA-F
HLA-DRA	MRPL17	VEPH1	GAL	TCTN3
HLA-DRA	MED11	PLAC1	HLA-H	TBC1D3B
LPPR4	VGLL3	SLC25A42	SHC4	AADAC
HLA-DRA	FLJ20021	TADA3	ELANE	LOC100506433
LPL	SEMA4B	CD59	KLHDC1	MCL1
HLA-DRA	CRMP1	KC6	RNF128	MDH1B
CT45A4	ALOX15B	S100A10	TMEM147	BANK1
HLA-DRA	DDB2	HDDC3	TMEM143	MIR33A
SAGE1	DEFB1	C12orf63	MSMB	HLA-J
UST	CD44	COL25A1	UBE2D4	RNASE10
TRIM31	HLA-DMA	SASH1	MDGA2	IFT140
GJB5	HLA-DMA	MIR1-1	NFX1	OR10W1
SLC43A3	HLA-DMA	ARL6IP6	PPIAL4A	PABPC4
NCAM2	HLA-DMA	AMOTL1	SERGEF	KLHL31
COL5A2	HLA-DMA	ISM2	KCNV1	C2orf68
AKT3	HLA-B	IDH2	CDY2B	HLA-H
ITPR1	CCNB3	IGF1R	PTPRM	GMPPA
LOC100506406	RNF122	LTBP1	HLA-F	PARP12
GNAT3	FRMD3	SUSD4	PTH1R	SHC3
TRIM31	TMEM194B	HLA-A	HLA-F	HLA-A
TRIM31	IGFBPL1	C16orf58	TBC1D3P2	RAD51B
TRIM31	RN5S269	STAT5A	HUS1B	ADRBK1
TRIM31	HLA-DMA	NKAPL	CLDN3	UQCRC1
CYP4F22	LINGO2	ZFP36L2	PLCB3	LOC375295
ITGB4	HIST1H4G	MIR381	HLA-H	ERBB3
TRIM31	RPLP0P2	DIRAS3	FHL2	HLA-J
TRIM31	SLCO5A1	ANK2	ZCCHC14	
LOC439990	ALS2CR8	CPM	MIR1258	
PLAUR	LOC100505666	TSTA3	MRPL12	
TRIM31	SGK1	L3MBTL3	SYNGAP1	
KCTD12	ITGB6	SLC25A10	LOC100131089	
KIAA0825	OR6T1	LUZP2	RASGRP3	
SCN9A	SHISA3	FAM114A2	LOC100506813	
NEUROG2	FLJ35776	DOT1L	PHPT1	
COL15A1	MIR4694	PLXND1	SYNGAP1	
LGALS3BP	SDC2	RPS6KA2	SNORD114-4	
SEMA6A	RNY4P3	C14orf79	P2RX4	

ERVH48-1	FBXO4	B3GALTL	GATA4

Genes upregulated by iAs exposure at >1.2 fold at FDR <0.05

TAF1L	SLC32A1	APOF	SNAP25
LINC00475	HECW1	OR6C65	SEMA3A
REM1	FGF12-AS1	TXNRD1	RNU7-47P
ZNF833P	ASTL	MIR4747	ALDH3A2
OPN3	LOC100506241	PDZK1P1	SPINK6
LOC100128420	LOC152024	FABP3P2	ALDH3A1
RBFOX3	TRBV3-1	PDZK1P1	TRIM16L
ZNF200	FECH	LINC00290	SH3GL2
MIR548I2	ZNF77	CTGF	ABCG2
SPG20OS	ZNF66P	ZNF469	GDF15
SNORA78	TYW1B	LOC644172	GCLM
OR7E5P	NKTR	FABP3	HMOX1
MRGPRX2	SNORD114-8	TTTY17A	SLC7A11
TPTE2P1	POU4F2	TTTY17C	
JPX	LOC100506021	TTTY17C	
TCL1A	ZBTB40	ALAS1	
TMEM212-IT1	RYR2	CDK12	
ZNF630-AS1	MIR124-1	LOC100505515	
OR8D2	LIMD1	KCNK18	
KAL1	GSTM2P1	MIR543	
GSTM2	LINC00481	GSR	
OSGIN1	LOC100507642	NUDT11	
MIR1266	MIR3179-3	TRIM16	
C1S	LNX1-AS2	LEP	
LOC339593	NT5C2	ZNF323	
SERPINB12	LOC100506271	FTL	
IGHV1-46	ACTRT2	NXPE3	
FGF19	RN5S265	WDFY1	
STYK1	RN5S439	MIR548H3	
CABYR	RASD1	ID2	
BHLHB9	RN5S237	ABCC2	
LINC00550	TMPRSS11GP	PIR	
CCDC170	OSTCP1	LOC344887	
FUZ	MIR548AH	KRTAP19-4	
MIR4518	LOC100009676	PSAT1	
FAM212B	LOC100505633	MIR4804	
CCND3	GNAS-AS1	DHRS2	
ANKK1	LOC339568	LOC100289211	
SPEF1	ZSCAN22	C1QTNF9-AS1	
OR11L1	ASIP	NEXN	
MIR4489	RN5S510	UGT1A5	
MOGAT3	LOC100130301	PASD1	
IZUMO2	NQO2	LOC283624	
LMOD1	SQSTM1	LOC100506899	

SDPR	LOC100505481	OR10P1
