# Supplementary material for: Inorganic Arsenic-induced cellular transformation is coupled with genome wide changes in chromatin structure, transcriptome and splicing patterns
Source: BMC Genomics. 2015 Mar 19;16(1):212. doi: 10.1186/s12864-015-1295-9 (PMC4371809; doi:10.1186/s12864-015-1295-9)
Supplement: Additional file 5: Table S2. — Heatmap showing the differential gene expression patterns of different solute carrier proteins and zinc-finger binding proteins modulated in the different experimental conditions. [file 12864_2015_1295_MOESM5_ESM.pdf]

Additional file 5: Table S2: Heatmap showing differential gene expression patterns of different solute carrier proteins and zinc-finger binding proteins in the different experimental conditions.

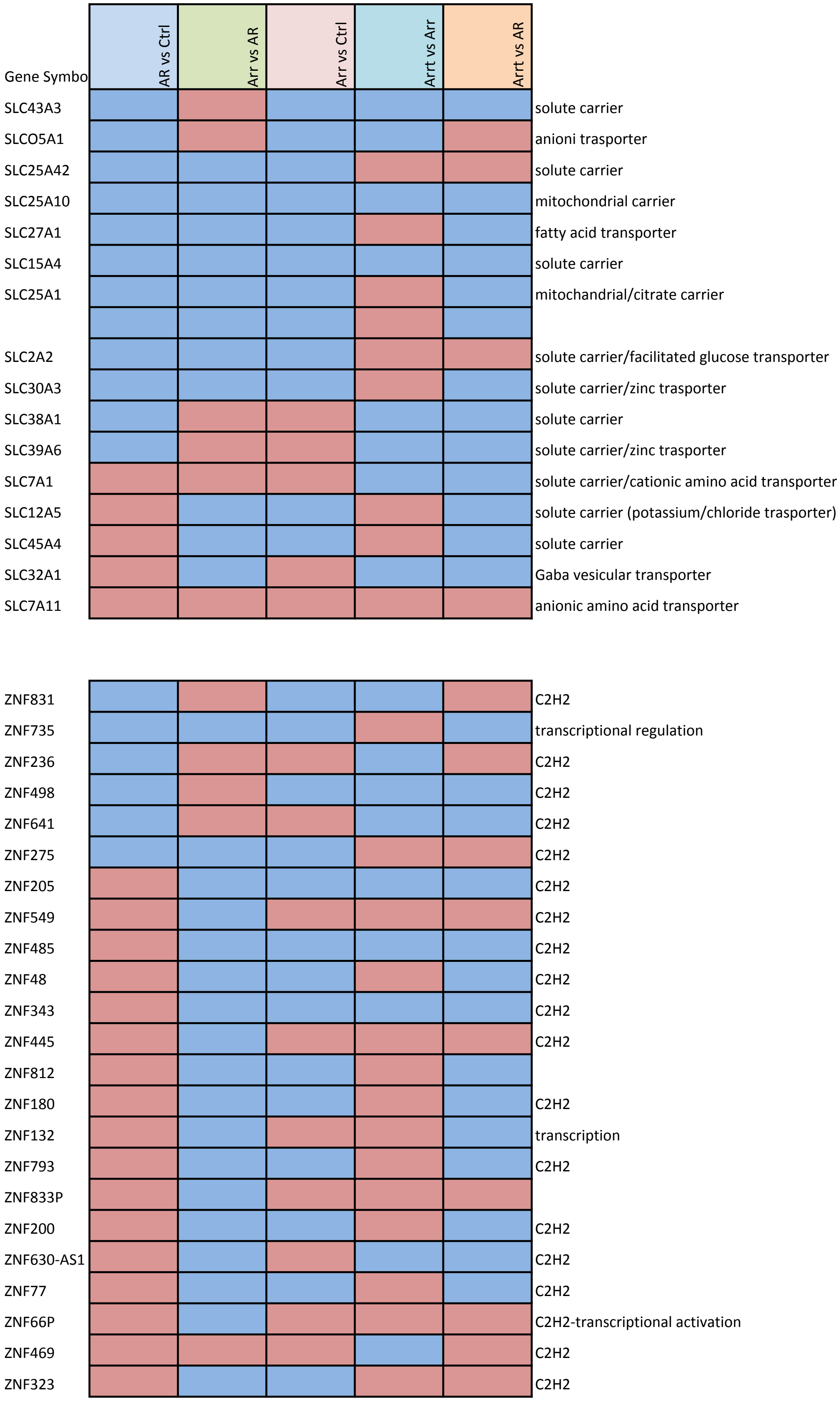

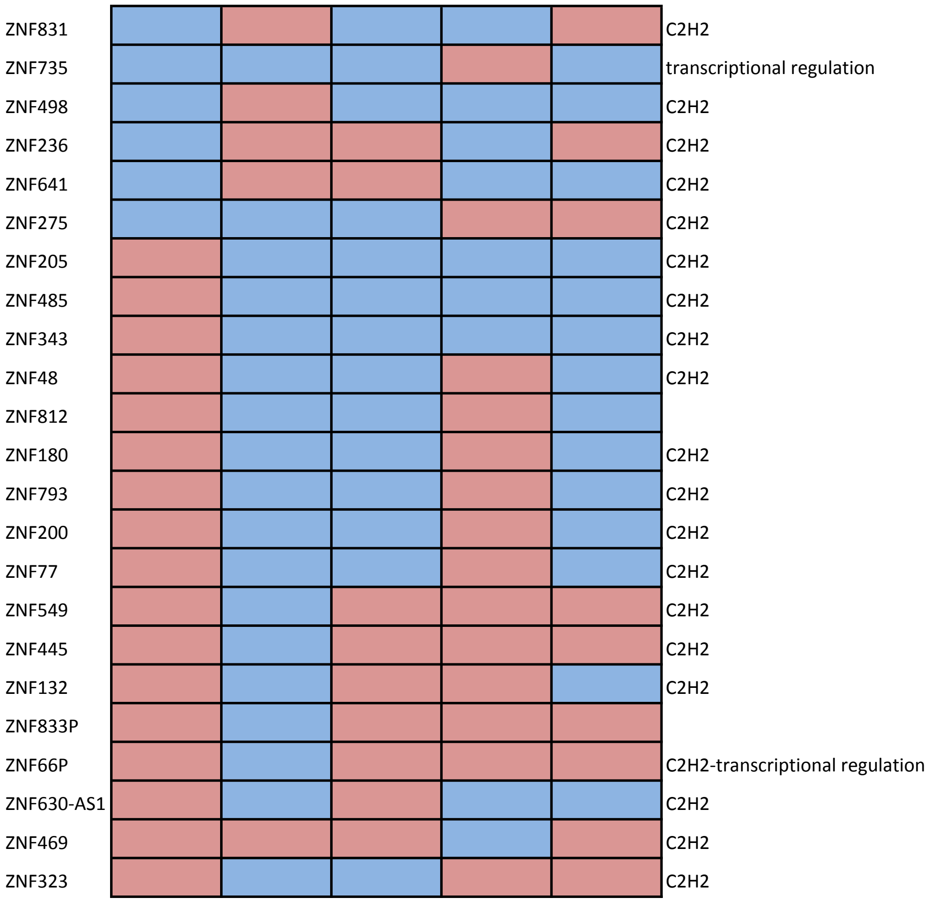
ZNF831
C2H2
ZNF735
transcriptional regulation
ZNF498
C2H2
ZNF236
C2H2
ZNF641
C2H2
ZNF275
C2H2
ZNF205
C2H2
ZNF485
C2H2
ZNF343
C2H2
ZNF48
C2H2
ZNF812
ZNF180
C2H2
ZNF793
C2H2
ZNF200
C2H2
ZNF77
C2H2
ZNF549
C2H2
ZNF445
C2H2
ZNF132
C2H2
ZNF833P
ZNF66P
C2H2-transcriptional regulation
ZNF630-AS1
C2H2
ZNF469
C2H2
ZNF323
C2H2
