# Supplementary material for: Inorganic Arsenic-induced cellular transformation is coupled with genome wide changes in chromatin structure, transcriptome and splicing patterns
Source: BMC Genomics. 2015 Mar 19;16(1):212. doi: 10.1186/s12864-015-1295-9 (PMC4371809; doi:10.1186/s12864-015-1295-9)
Supplement: Additional file 6: Table S3. — Transcription factors whose binding sites were detected at the promoters of iAs-target genes. [file 12864_2015_1295_MOESM6_ESM.pdf]

Additional File 6: Table S3: Transcription factors whose binding sites were detected at promoters of iAs-target genes.

Transcription factor	# Genes	Genes	P-value
E12	48	UST, LIMD1, FABP3, SDPR, LTBP1, SIDT1, NPAS2, ITGA6, PTP4A3, SEMA6A, LTBP3, LGALS3BP, PABPC4, PLCB3, PRKCH, ADRBK1, RNF128, IGF1R, SQSTM1, SLC7A11, SLC25A10, LPHN1, METTL8, ERBB3, CKB, CLDN3, ITGB6, ETV4, POU4F2, HDDC3, CDYL2, STAG1, TSTA3, ANK2, SNAP25, SERGEF, ARHGAP24, ZFP3, SASH1, SH3GL2, NCAM2, LPL, ITGB4, CRMP1, F2RL1, SEMA3A, RNF122, SEMA4B	5.42e-07
FOXO4	38	NPAS2, MFAP5, ID2, RASD1, RASGRP3, SEMA6A, SLC7A11, SQSTM1, IGF1R, NPFFR2, LPHN1, SGK1, ERBB3, CKB, ETV4, FAM134C, POU4F2, DUSP1, ZCCHC14, HECW1, STAG1, PTPRM, VGLL3, C12orf63, CD36, NT5C2, SASH1, AKT3, CDH5, ITPR1, SLC27A1, ITGB4, NUDT11, NEUROG2, CRMP1, SEMA3A, RNF122, SEMA4B	5.94e-05
LEF1	36	UST, SDPR, AMOTL1, PTH1R, KCTD12, SEMA6A, LTBP3, PLCB3, SLC7A11, IGF1R, SHC3, SLC25A10, ERBB3, CKB, ETV4, CDK12, DUSP1, TXNRD1, FTL, STAG1, CSNK1E, ANK2, VGLL3, ARHGAP24, FECH, ARHGEF19, SLC32A1, AKT3, CDH5, COL15A1, SLCO5A1, CRMP1, SEMA3A, RNF122, L1CAM, SEMA4B	9.41e-05
AACTTT_unknown	32	UST, SDPR, TDP1, LTBP1, AMOTL1, B3GALTL, PSAT1, STAG1, SIDT1, NPAS2, ID2, SEMA6A, SERGEF, PLCB3, RPS6KA2, ADRBK1, RNF128, IGF1R, SH3GL2, SHC3, NCAM2, AKT3, COL25A1, CDH5, ITGB4, ETV4, NF122, F2RL1, SEMA3A, ZFP36L2, CDK12, POU4F2	0.0014
MAZ	36	UST, AMOTL1, PARP12, PTH1R, NPAS2, MGP, DPAGT1, GDF15, PLCB3, ARL6IP6, PRKCH, CTGF, IGF1R, COL25A1, LPHN1, RAPGEFL1, ERBB3, ALS2CR8, MYCN, CD44, ETV4, CDK12, STAG1, COL5A2, PLAUR, CSNK1E, RYR2, SNAP25, ARHGAP24, HLA-DMA, ARHGEF19, SLC32A1, NCAM2, ITPR1, FRMD3, CRMP1	0.0016
FREAC2	20	UST, PTH1R, VGLL3, ANK2, FHL2, ID2, PTP4A3, RASD1, KCTD12, TMEM147, SNAP25, PRKCH, ABTB1, RNF128, CTGF, SQSTM1, IGF1R, RAPGEFL1, ZFP36L2, DUSP1	0.00016
GATA4	9	COL15A1, LPHN1, ID2, SLCO5A1, RAPGEFL1, NEUROG2, SEMA3A, NEXN, POU4F2	0.0051
GGYGTGNY	15	TXNRD1, HACL1, PARP12, CDH5, TMEM143, DPAGT1, RAPGEFL1, ITGB4, CLDN3, CD44, ETV4, CRMP1, TTLL1, SEMA6A, PLCB3	0.0051
NFAT	30	HDDC3, LTBP1, AMOTL1, STAG1, NPAS2, MGP, VGLL3, ID2, RASGRP3, PLAC1, SNAP25, SEMA6A, EDNRA, PRKCH, HLA-B, NT5C2, SASH1, RNF128, CTGF, S100A10, LRRC6, AKT3, CDH5, ITPR1, SGK1, LPL, LEP, ERBB3, KIAA0825, SEMA3A	0.0051
ETS2	20	HDDC3, SDPR, GATA4, PTH1R, NPAS2, RAC1, RASGRP3, ARHGDIB, LTBP3, PRKCH, NT5C2, RNF128, SHC3, CDH5, ALDH3A1, ITPR1, SGK1, CRMP1, RNF122, CDK12	0.0071
