# Supplementary material for: Inorganic Arsenic-induced cellular transformation is coupled with genome wide changes in chromatin structure, transcriptome and splicing patterns
Source: BMC Genomics. 2015 Mar 19;16(1):212. doi: 10.1186/s12864-015-1295-9 (PMC4371809; doi:10.1186/s12864-015-1295-9)
Supplement: Additional file 7: Table S4. — Pathways involved by the genes targeted by iAs. Detailed analyses of the iAs-targeted genes and their association with cancer (analyzed using GSEA). [file 12864_2015_1295_MOESM7_ESM.pdf]

Additional File 7: Table S4: Pathways involved by the genes targeted by iAs

Pathway	Number of genes
Gonadotropin-releasing hormone receptor pathway	216
Angiogenesis	77
Apoptosis signaling pathway	72
p53 pathway	70
Alzheimer disease-presenilin pathway	70
Huntington disease	60
Blood coagulation	59
Inflammation mediated by chemokine and cytokine signaling pathway	58
Wnt signaling pathway	49
Toll receptor signaling pathway	46
Integrin signalling pathway	46
T cell activation	45
Ras Pathway	39
B cell activation	37
Parkinson disease	37
p38 MAPK pathway	36
Interleukin signaling pathway	36
PDGF signaling pathway	36
PI3 kinase pathway	35
p53 pathway feedback loops 2	32
Alzheimer disease-amyloid secretase pathway	31
FAS signaling pathway	31
Ionotropic glutamate receptor pathway	29
EGF receptor signaling pathway	28
Nicotine pharmacodynamics pathway	27
Endothelin signaling pathway	27
Dopamine receptor mediated signaling pathway	27
Heterotrimeric G-protein signaling pathway-Gi alpha and Gs alpha mediated pathway	27
FGF signaling pathway	26
Transcription regulation by bZIP transcription factor	25
VEGF signaling pathway	25
Notch signaling pathway	23
De novo purine biosynthesis	23
Plasminogen activating cascade	22
DPP-SCW signaling pathway	21
Angiotensin II-stimulated signaling through G proteins and beta-arrestin	21
Heterotrimeric G-protein signaling pathway-Gq alpha and Go alpha mediated pathway	21
Hedgehog signaling pathway	20
TGF-beta signaling pathway	20
Oxidative stress response	20
Cytoskeletal regulation by Rho GTPase	20
DPP signaling pathway	19
General transcription regulation	19
Vitamin B6 metabolism	19
BMP/activin signaling pathway-drosophila	19
SCW signaling pathway	18
Insulin/IGF pathway-protein kinase B signaling cascade	18
GBB signaling pathway	17
Toll pathway-drosophila	17
Cadherin signaling pathway	16
General transcription by RNA polymerase I	16
Heterotrimeric G-protein signaling pathway-rod outer segment phototransduction	15
Heme biosynthesis	15
Axon guidance mediated by Slit/Robo	14
Hypoxia response via HIF activation	14

Circadian clock system	14
Activin beta signaling pathway	14
Axon guidance mediated by semaphorins	14
ALP23B signaling pathway	13
p53 pathway by glucose deprivation	13
Nicotinic acetylcholine receptor signaling pathway	13
MYO signaling pathway	13
Pentose phosphate pathway	13
Opioid prodynorphin pathway	12
Metabotropic glutamate receptor group III pathway	12
ATP synthesis	12
Cell cycle	12
Synaptic vesicle trafficking	12
Muscarinic acetylcholine receptor 1 and 3 signaling pathway	12
Adrenaline and noradrenaline biosynthesis	12
Insulin/IGF pathway-mitogen activated protein kinase kinase/MAP kinase cascade	12
Axon guidance mediated by netrin	11
Tetrahydrofolate biosynthesis	11
Vasopressin synthesis	11
Muscarinic acetylcholine receptor 2 and 4 signaling pathway	11
Opioid proenkephalin pathway	11
P53 pathway feedback loops 1	11
De novo pyrimidine deoxyribonucleotide biosynthesis	10
Metabotropic glutamate receptor group I pathway	10
Interferon-gamma signaling pathway	10
Pyruvate metabolism	10
Enkephalin release	10
De novo pyrimidine ribonucleotides biosythesis	10
mRNA splicing	10
Glycolysis	10
