# Supplementary material for: Inorganic Arsenic-induced cellular transformation is coupled with genome wide changes in chromatin structure, transcriptome and splicing patterns
Source: BMC Genomics. 2015 Mar 19;16(1):212. doi: 10.1186/s12864-015-1295-9 (PMC4371809; doi:10.1186/s12864-015-1295-9)
Supplement: Additional file 8: Table S5. — Genes altered by iAs and their association with various cancer modules. [file 12864_2015_1295_MOESM8_ESM.pdf]

Additional File 8: Table S5: Genes altered by iAs and their association with various cancer module

Table S5A: Cancer modules determined using KEGG analyses

Gene Set Name	# of genes in Gene Set (k)	Description	# Genes in Overlap (K)	k/K	p-value	FDR q-value
MODULE_117	731	Genes in the cancer module 117	38	0.0520	3.49E-12	1.5E-9
MODULE_55	834	Genes in the cancer module 55	34	0.0408	2.33E-8	5.01E-6
MODULE_88	838	Genes in the cancer module 88	32	0.0382	2.6E-7	3.7E-5
MODULE_293	12	Genes in the cancer module 293	5	0.4167	3.43E-7	3.7E-5
MODULE_176	230	Genes in the cancer module 176	15	0.0652	7.91E-7	6.09E-5
MODULE_143	14	Genes in the cancer module 143	5	0.3571	8.48E-7	6.09E-5
MODULE_220	333	Genes in the cancer module 220	18	0.0541	1.01E-6	6.21E-5
MODULE_2	384	Genes in the cancer module 2	19	0.0495	1.89E-6	9.32E-5
MODULE_11	540	Genes in the cancer module 11	23	0.0426	2.1E-6	9.32E-5
MODULE_60	425	Genes in the cancer module 60	20	0.0471	2.19E-6	9.32E-5

Table S5b: Detailed analyses of the iAs-targeted genes and their association with cancer (analyzed using GSEA).

**Genes**	**CANCER MODULES**	**117**	**55**	**88**	**293**	**176**	**143**	**220**	**2**	**11**	**60**
AADAC		✔	✔	✔		✔					
MSMB		✔	✔	✔							
TFPI2		✔				✔					
ARHGDIB		✔						✔			
CDH5		✔							✔		✔
DIRAS3		✔									
ALOX15B		✔									
CTSZ		✔									
GNRH1		✔									
OTC		✔									
POU4F2		✔									
REM1		✔									
NCAM2		✔									
SERPINI2		✔									
CNTN5		✔									
MMP20		✔									
CLCA2		✔									
KLRAP1		✔									
CCL19		✔									
PRKCH		✔									
ALDH3A1		✔									
CNR1		✔									
CDH6		✔									
BLK		✔									
PLCD1		✔									
ASPA		✔									
CD36		✔									
ELANE		✔									
CPM		✔									
SLC12A5		✔									
RASGRP3		✔									
ACTN2		✔									
RYR2		✔									
TJP3		✔									
RENBP		✔									
LRRC6		✔									
GATA4		✔									
SLC2A2		✔									
EPAS1		✔	✔	✔		✔		✔		✔	✔
ALDH3A2			✔	✔		✔		✔			

C1S	✔	✔		✔			✔		
DHRS2	✔	✔		✔					✔
COL5A2	✔	✔		✔					
DEFB1	✔	✔		✔					
PTH1R	✔	✔		✔					
L1CAM	✔	✔				✔	✔	✔	
HOXB2	✔	✔				✔			
TCL1A	✔	✔				✔			
CCND2	✔	✔					✔	✔	✔
PTP4A3	✔	✔					✔	✔	✔
ITGA6	✔	✔					✔	✔	
SNAP25	✔	✔					✔	✔	
ERBB3	✔	✔					✔	✔	
HLA-DMA	✔	✔					✔		✔
GDF15	✔	✔					✔		
TIMM17B	✔	✔						✔	✔
NECAB3	✔	✔						✔	✔
TRIM16	✔	✔						✔	✔
HMOX1	✔	✔							✔
ALAS1	✔	✔							✔
SLC7A11	✔	✔							
RIBC2	✔	✔							
CLDN3	✔	✔							
CYP2C19	✔	✔							
ASIP	✔	✔							
HBZ	✔	✔							
MGP	✔					✔			
SLC25A1	✔						✔		
GCLM	✔								
APOC3	✔								
LPL		✔					✔	✔	✔
FCER1G		✔							✔
HLA-A			✔		✔				
HLA-G			✔		✔				
HLA-DRA			✔		✔				
HLA-B			✔		✔				
HLA-F			✔		✔				
CRMP1				✔		✔		✔	
COL15A1				✔			✔		✔
OMG				✔				✔	
FHL2				✔					✔
KLRC1				✔					

RPS6KA2	✔				
KAL1		✔		✔	
CTGF		✔			
TBX2		✔			
GSTP1		✔			
GJB5		✔			
KRT32		✔			
ADAM23		✔			
SH3GL2		✔			
SEMA3A		✔			
NHLH2		✔			
SLC39A6			✔	✔	
NOTCH3			✔		✔
ITGB4			✔		✔
TRIB2			✔		
CD44			✔		
LTBP1			✔		
PARD6A				✔	✔
PTPRZ1				✔	
ITPR1				✔	
GABRG2				✔	
IQSEC1				✔	
FAM131A				✔	
SGK1				✔	
LPHN1				✔	
PLXND1					✔
