# Supplementary material for: Inorganic Arsenic-induced cellular transformation is coupled with genome wide changes in chromatin structure, transcriptome and splicing patterns
Source: BMC Genomics. 2015 Mar 19;16(1):212. doi: 10.1186/s12864-015-1295-9 (PMC4371809; doi:10.1186/s12864-015-1295-9)
Supplement: Additional file 9: Figure S4. — Genes common in both iAs-T and iAs-Rev cells. These analyses show genes that did not revert to NT conditions. Also shown are the functions of some of these genes. [file 12864_2015_1295_MOESM9_ESM.pdf]

Additional File 9: Figure S4

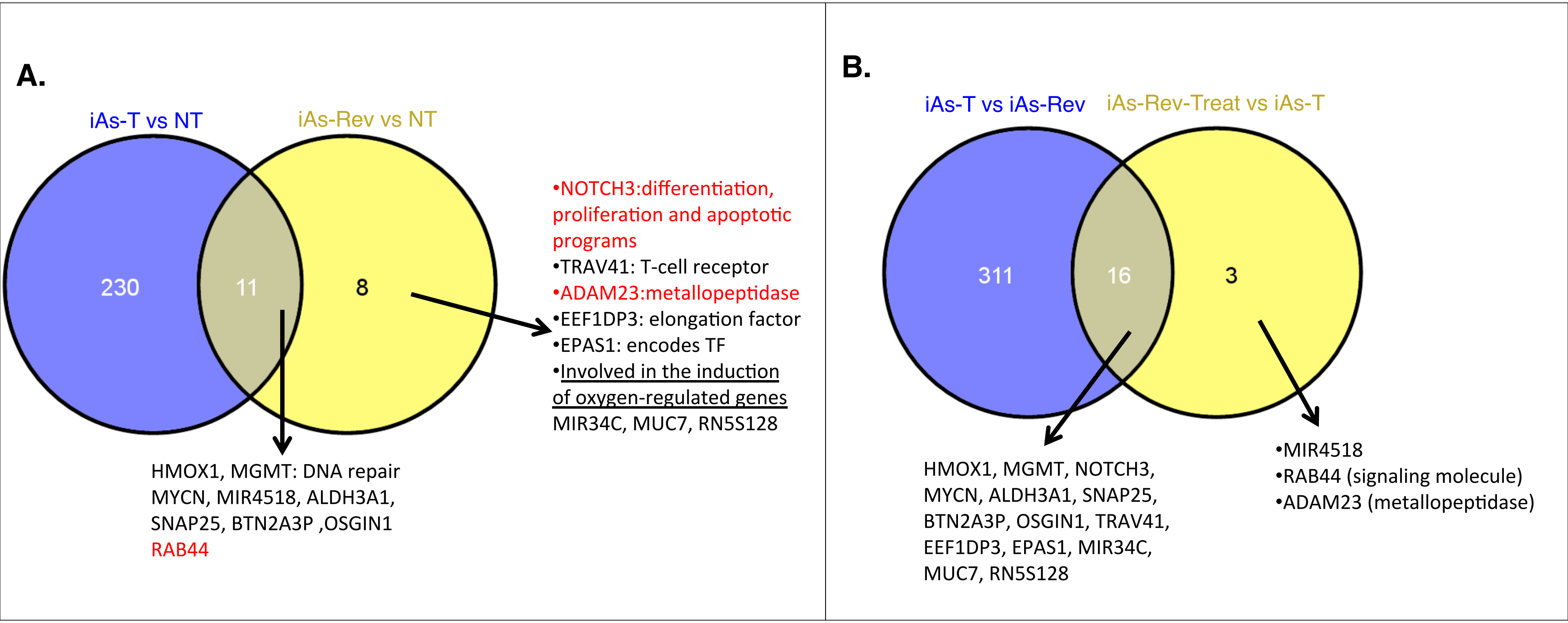

Additional File 9: Figure S4: Genes common in both iAs-T and iAs-Rev cells. These analyses show genes that did not revert to NT conditions. Also shown are the functions of some of these genes.
