# Supplementary material for: Inorganic Arsenic-induced cellular transformation is coupled with genome wide changes in chromatin structure, transcriptome and splicing patterns
Source: BMC Genomics. 2015 Mar 19;16(1):212. doi: 10.1186/s12864-015-1295-9 (PMC4371809; doi:10.1186/s12864-015-1295-9)
Supplement: Additional file 10: Figure S5. — Heatmap of the expression pattern of genes common in iAs-T, iAs-rev and iAs-rev-reTreat cells. Red: indicates upregulation and green indicates down regulation. From dark green to light green = low fold change downregulation to more down regulation. Dark red to light red = degree of upregulation: small to high upregulation. [file 12864_2015_1295_MOESM10_ESM.pdf]

Additional File 10: Figure S5

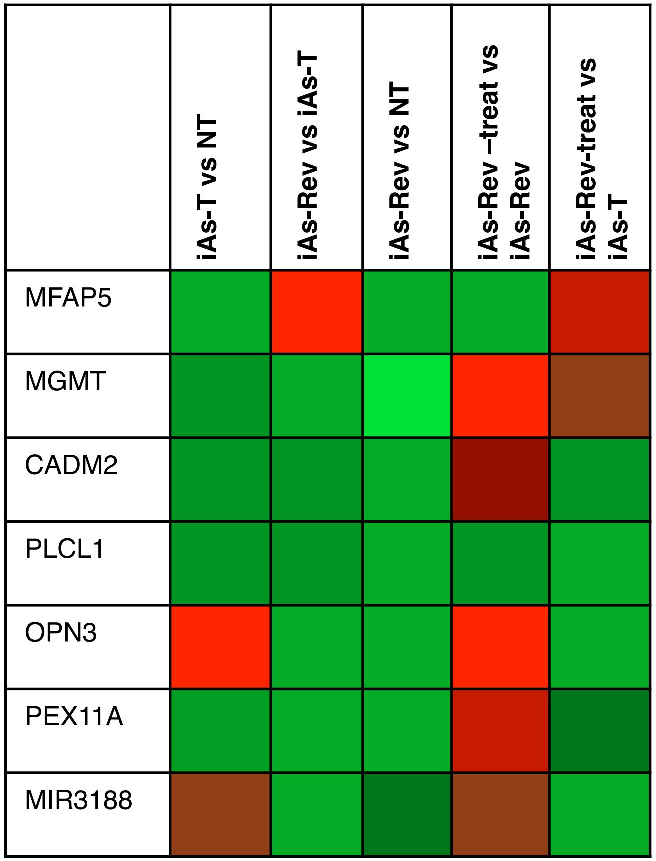

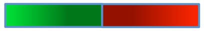

Additional File 10: Figure S5: Heatmap of the expression pattern of genes common in iAs-T, iAs-rev and iAs-rev-reTreat cells. Red: indicates upregulation and green indicates down regulation. From dark green to light green = low fold change downregulation to more down regulation. Dark red to light red = less upregulation to high upregulation.
