# Supplementary material for: Inorganic Arsenic-induced cellular transformation is coupled with genome wide changes in chromatin structure, transcriptome and splicing patterns
Source: BMC Genomics. 2015 Mar 19;16(1):212. doi: 10.1186/s12864-015-1295-9 (PMC4371809; doi:10.1186/s12864-015-1295-9)
Supplement: Additional file 11: Figure S6. — Protein interaction map of altered iAs-target genes. The bioinformatics STRING database (version 9.1) was used to generate a protein interaction map with known and predicted protein associations that include direct physical and indirect functional protein linkages of microarray identified iAs-target genes at protein level from Additional file 10: Figure S5). Shown also are the interactors based on evidence (left side) and confidence level (right side). i) Evidence view: different line colors represent the types of evidence for the association. Green: neighborhood; red: gene fusion; blue: co-occurrence; black/grey: co-expression; pink: experiments; teal: databases; Pea green: textmining; purple: homology ii) Confidence view: Thicker lines represent stronger associations. [file 12864_2015_1295_MOESM11_ESM.pdf]

# Additional File 11: Figure S6

## A. MFAP5

*i) Evidence*

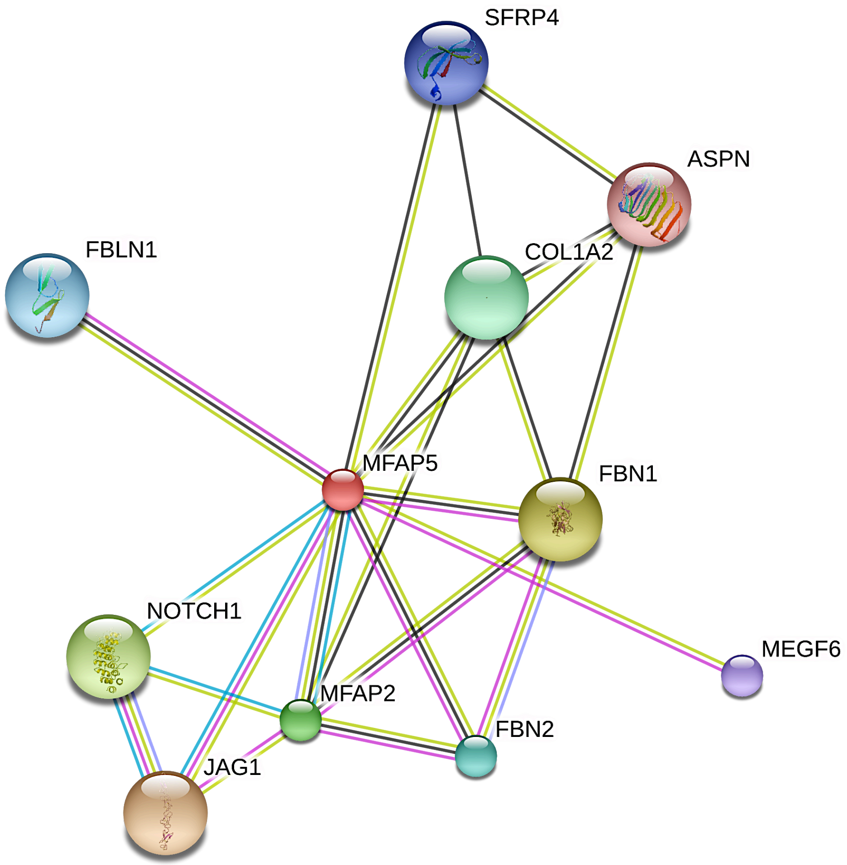


*ii) Confidence*

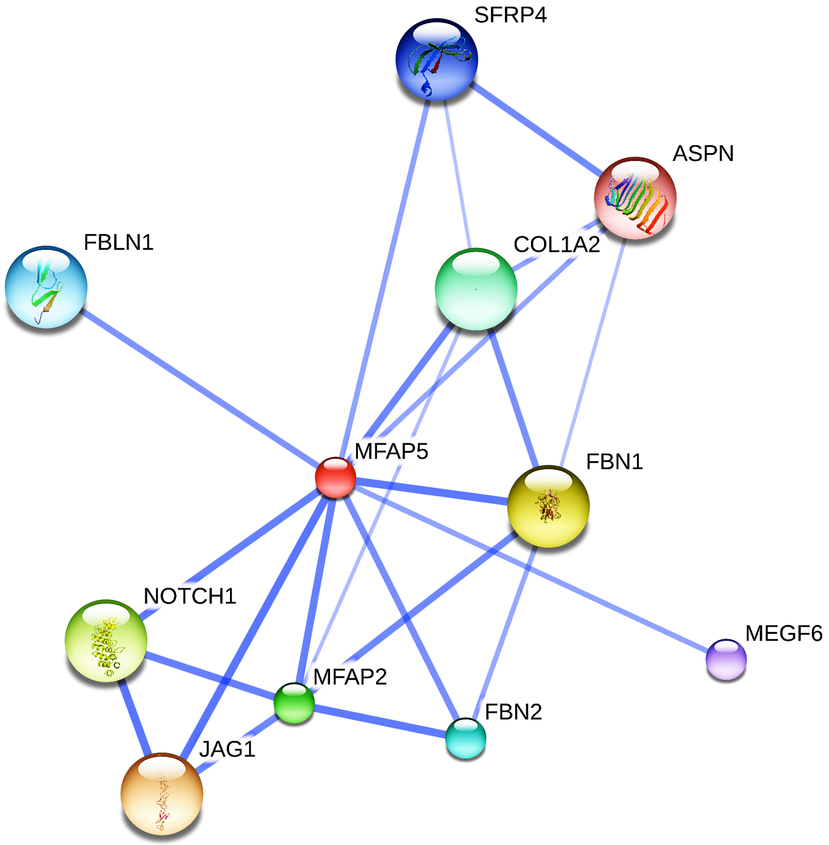

**B: MGMT**

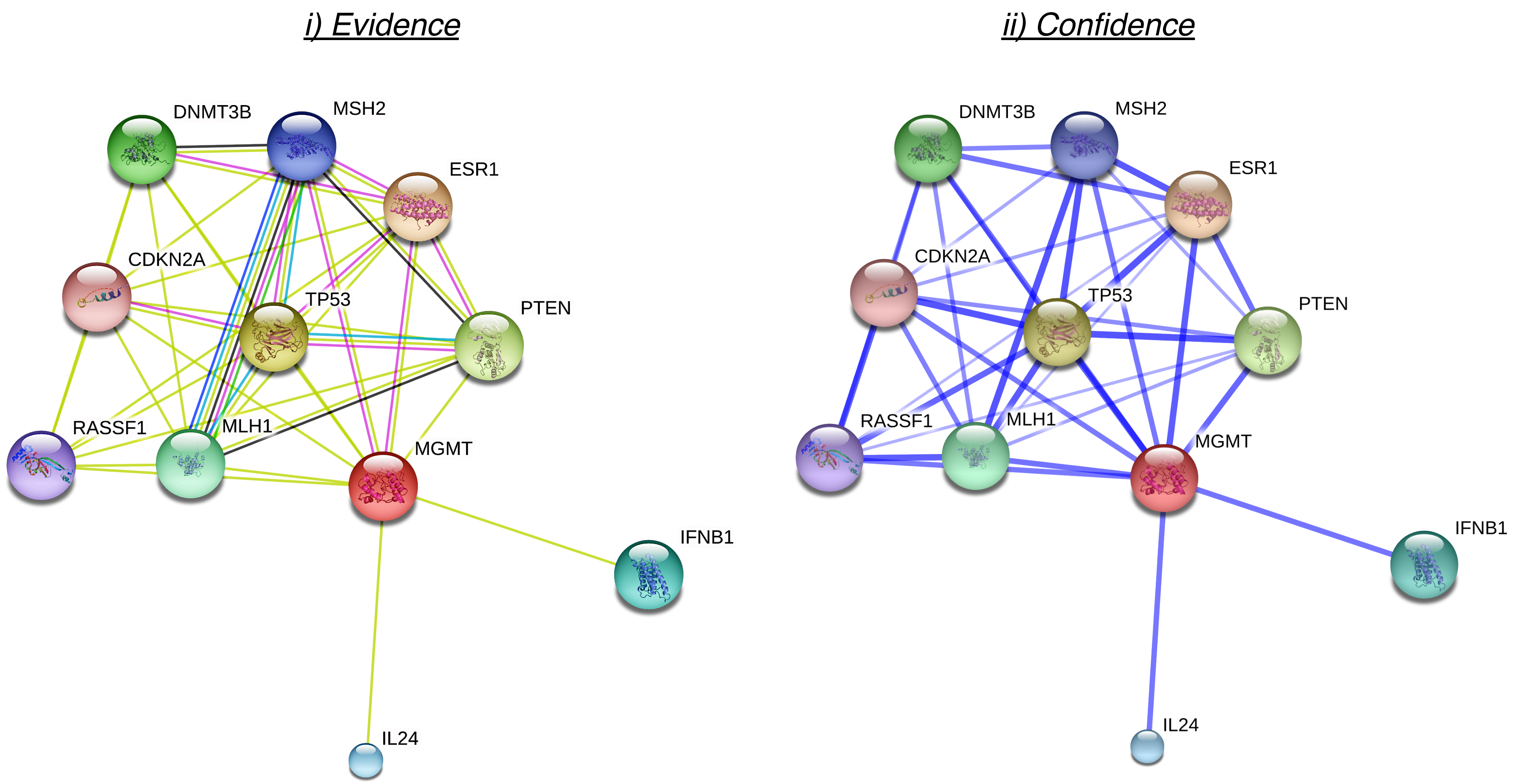
i) Evidence
ii) Confidence
DNMT3B
MSH2
ESR1
CDKN2A
TP53
PTEN
RASSF1
MLH1
MGMT
IFNB1
IL24
DNMT3B
MSH2
ESR1
CDKN2A
TP53
PTEN
RASSF1
MLH1
MGMT
IFNB1
IL24

**C: OPN3**

*i) Evidence*

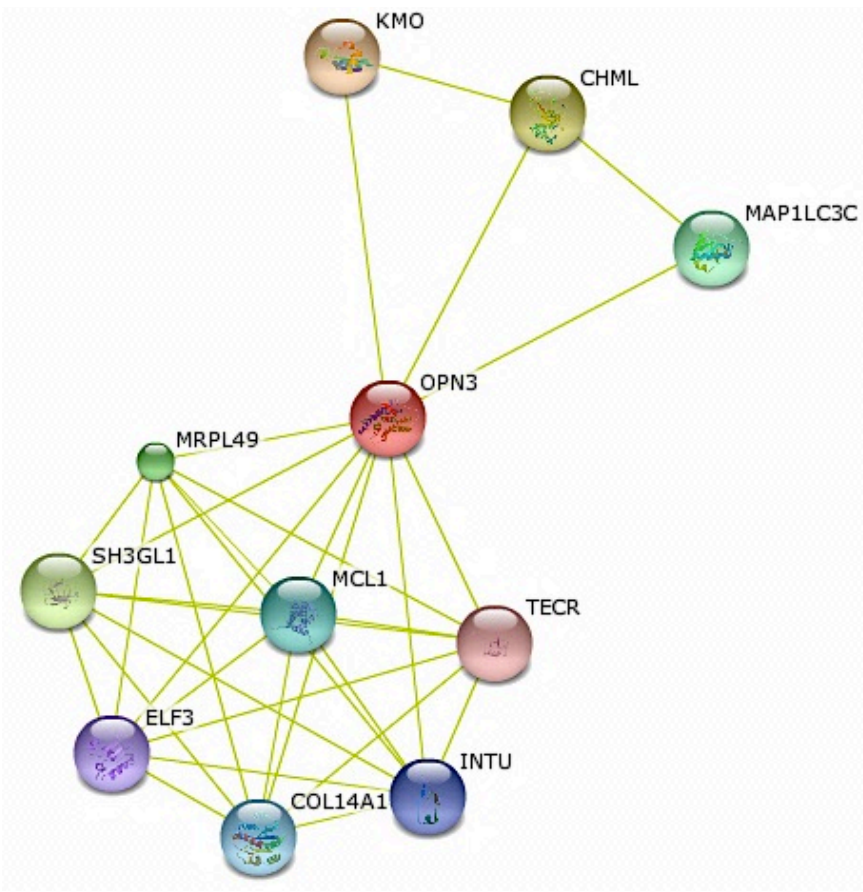


*ii) Confidence*

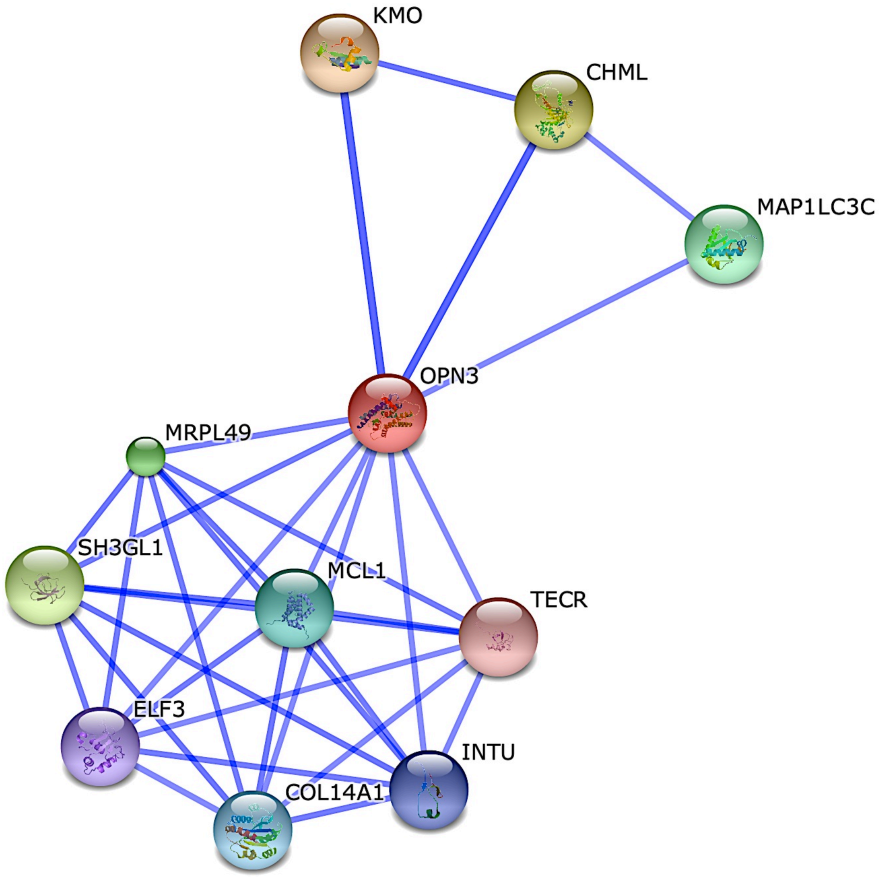

**D: PEX11A**

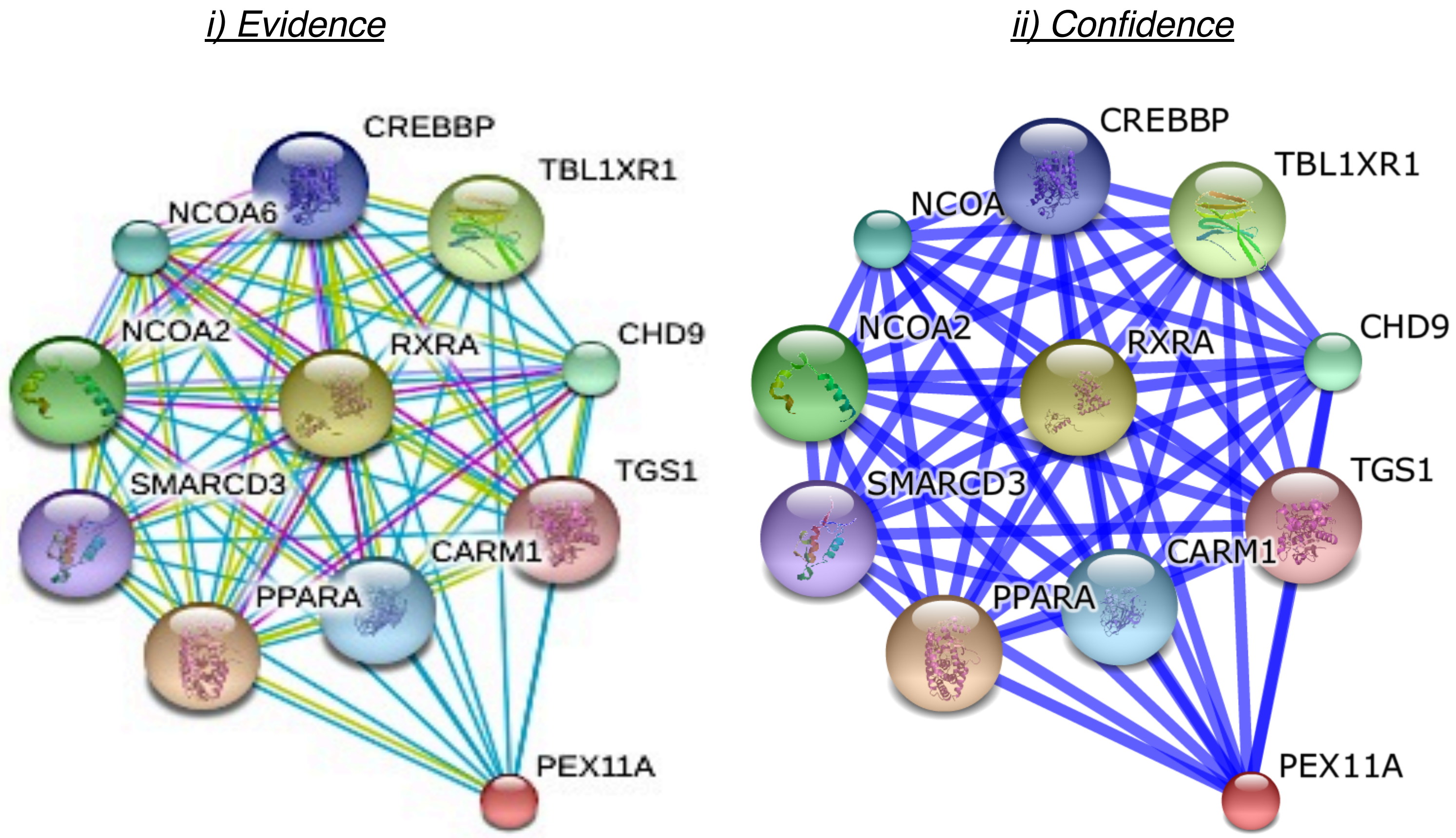
i) Evidence
CREBBP
TBL1XR1
NCOA6
NCOA2
RXRA
CHD9
TGS1
SMARCD3
CARM1
PPARA
PEX11A
ii) Confidence
CREBBP
TBL1XR1
NCOA
NCOA2
RXRA
CHD9
TGS1
SMARCD3
CARM1
PPARA
PEX11A

**E: CADM2**

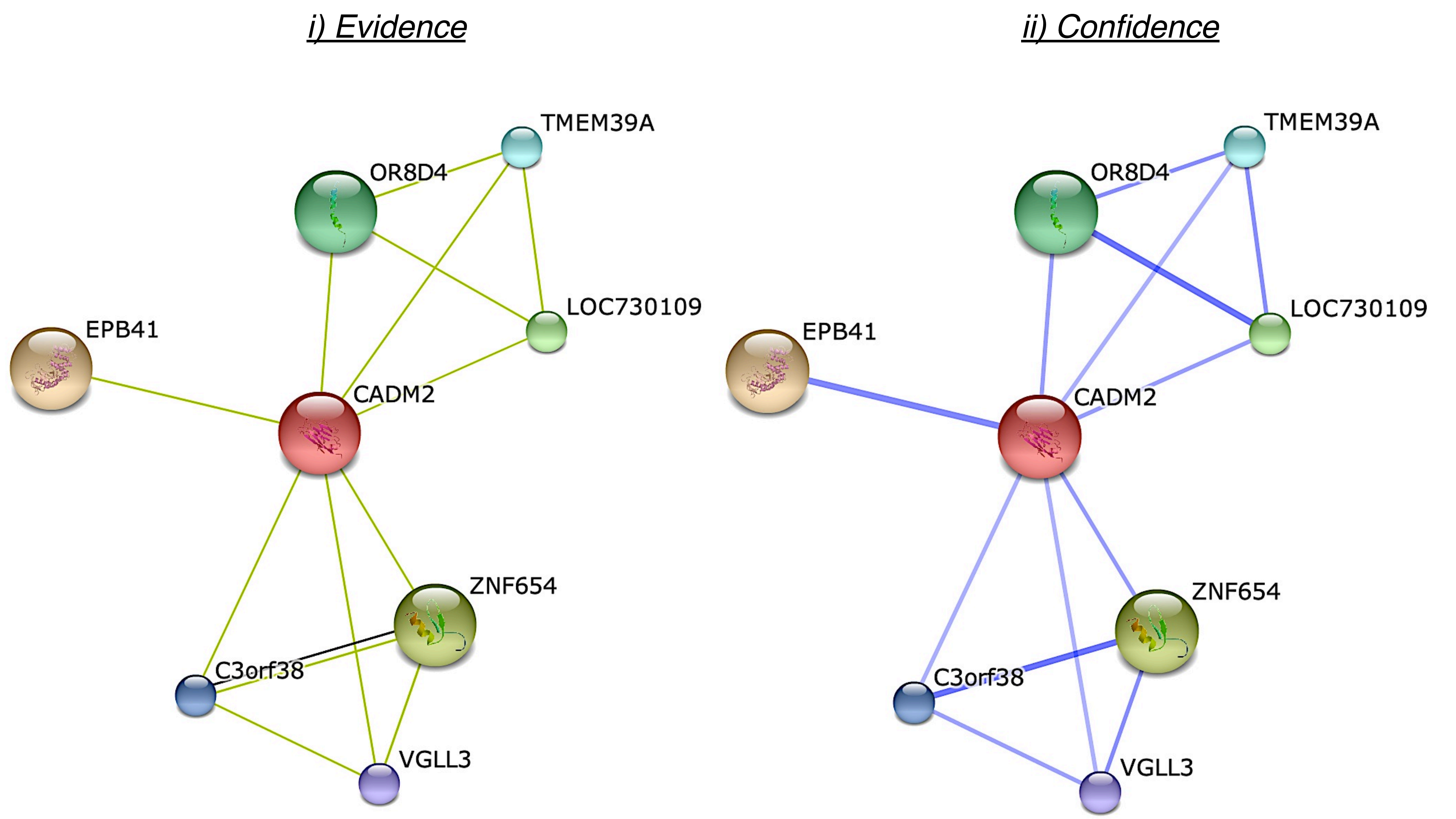

i) Evidence
ii) Confidence
TMEM39A
OR8D4
LOC730109
EPB41
CADM2
ZNF654
C3orf38
VGLL3
TMEM39A
OR8D4
LOC730109
EPB41
CADM2
ZNF654
C3orf38
VGLL3

**Additional File 11: Figure S6:** Protein interaction map of altered *iAs*-target genes. The bioinformatics STRING database (version 9.1 was used to generate a protein interaction map with known and predicted protein associations that include direct physical and indirect functional protein linkages of microarray identified iAs-target genes at protein level from Fig S5). Shown also are the interactors based on evidence (left side) and confidence level (right side). i) Evidence view: different line colors represent the types of evidence for the association. Green: neighborhood; red: gene fusion; blue: co-occurrence; black/grey: co-expression; pink: experiments; teal: databases; Pea green: textmining; purple: homology ii) Confidence view: Thicker lines represent stronger associations.
