# Supplementary material for: Inorganic Arsenic-induced cellular transformation is coupled with genome wide changes in chromatin structure, transcriptome and splicing patterns
Source: BMC Genomics. 2015 Mar 19;16(1):212. doi: 10.1186/s12864-015-1295-9 (PMC4371809; doi:10.1186/s12864-015-1295-9)
Supplement: Additional file 12: Table S6. — Genes with iAs-mediated alternatively spliced events. [file 12864_2015_1295_MOESM12_ESM.pdf]

Additional File 12: Table S6: Genes with iAs-mediated alternatively spliced events

**DOWNREGULATED AND SPLICED**			
UCA1	MFAP5	ARHGDIB	MGP
CD36	LPL	SAGE1	UST
GJB5	SLC43A3	NCAM2	COL5A2
AKT3	ITPR1	GNAT3	CYP4F22
ITGB4	PLAUR	KCTD12	SCN9A
COL15A1	LGALS3BP	SEMA6A	NPAS2
EDNRA	L1CAM	ETV4	MRPL17
SEMA4B	ALOX15B	RNF122	FRMD3
HLA-DMA	LINGO2	ALS2CR8	SDC2
TTLL1	SIDT1	RIBC2	COL25A1
SASH1	ARL6IP6	ISM2	IDH2
SUSD4	C16orf58	STAT5A	DIRA3
ANK2	CPM	LUZP2	DOTL1L
PLXND1	RPS6KA2	TSPAN18	SHC4
ELANE	MDGA2	KCNV1	CDY2B
HLA-F	PTH1R	FHL2	P2RX4
GATA4	IFT140	KLHL31	SHC3
UQCRC1	NUDT8	CDH5	NPFFR2
SLITRK4	PDZD4	MKX	FCER1G
PTK6	PRKCG	SEC22C	NAT6
NEGR1-IT1			
**UPREGULATED AND SPLICED**			
DOK2	THAP3	PDCD6	NOSTRIN
PRR23A	PYCARD	GZMH	STEAP2
T1CAM1	SLC45A4	COL24A1	C1S
FAM212B	RYR2	LIMD1	SQSTM1
ZNF469	FABP3	TRIM16	ZNF323
DHRS2	UGAT1A5	SEMA3A	ALDH3A1
SH3GL2	ABCG2	HMOX1	
