# Supplementary material for: Inorganic Arsenic-induced cellular transformation is coupled with genome wide changes in chromatin structure, transcriptome and splicing patterns
Source: BMC Genomics. 2015 Mar 19;16(1):212. doi: 10.1186/s12864-015-1295-9 (PMC4371809; doi:10.1186/s12864-015-1295-9)
Supplement: Additional file 13: Table S7. — MicroRNAs altered during arsenite exposure. [file 12864_2015_1295_MOESM13_ESM.pdf]

Additional File 13: Table S7: MicroRNAs altered during arsenite exposure

Comparison	Up-Regulated	Down-Regulated
**IAs-T vs. NT**	MIR100HG, MIR122, MIR127, MIR2117, MIR3150B, MIR3179-1, MIR3179-2, MIR3179-3, MIR320D2, MIR3591, MIR4257, MIR4747, MIR4804, MIR493, MIR543, MIR921	MIR4773-1, MIR3202-1, MIR4694, MIR3170
**iAs-rev vs. NT**	MIR1263, MIR2116, MIR4804, MIR512-1, MIR512-2, MIR543, MIR548AL, MIR548C, MIR548F2, MIR548I2, MIR548Z, MIR598	MIR101-1, MIR1-1, MIR1185-1, MIR1266, MIR31, MIR4637, MIR4779
**iAs-rev-reTreat vs. NT**	MIR1207, MIR1294, MIR2116, MIR320C1, MIR329-2, MIR3622B, MIR3649, MIR3690, MIR3936, MIR4417, MIR4476, MIR4489, MIR4518, MIR4535, MIR4652, MIR4682, MIR4731, MIR4804, MIR500A, MIR548AJ2, MIR548H3, MIR548I2, MIR548Q, MIR595, MIR598, MIR603, MIR708	MIR1-1, MIR133B, MIR3138, MIR3977, MIR4773-1, MIR583
